# Data release: targeted systematic literature search for tick and tick-borne pathogen distributions in six countries in sub-Saharan Africa from 1901 to 2020

**DOI:** 10.1186/s13071-023-06086-4

**Published:** 2024-02-22

**Authors:** Abigail A. Lilak, David B. Pecor, Graham Matulis, Alexander M. Potter, Rachel N. Wofford, Mary F. Kearney, Stephanie Mitchell, Fatima Jaradat, Arisa Kano, Dawn M. Zimmerman, James M. Hassell, Bersissa Kumsa, Maureen Kamau, Yvonne-Marie Linton, Michael E. von Fricken

**Affiliations:** 1 One Health Center of Excellence, Emerging Pathogens Institute, University of Florida, 2055 Mowry Road, Gainesville, FL 32611 USA; 2 Department of Environmental & Global Health, University of Florida, Gainesville, FL USA; 3 Walter Reed Biosystematics Unit (WRBU), Smithsonian Museum Support Center, Suitland, MD USA; 4 Department of Entomology, Smithsonian Institution—National Museum of Natural History, Washington, DC USA; 5 One Health Branch, Walter Reed Army Institute of Research (WRAIR), Silver Spring, Maryland, USA; 6 George Mason University, Fairfax, VA USA; 7 University of Michigan, Ann Arbor, MI USA; 8 Yale University, New Haven, CT USA; 9 Department of Pathology & Parasitology, College of Veterinary Medicine and Agriculture, Addis Ababa University, Bishoftu, Ethiopia; 10 Mpala Research Center, Nanyuki, Kenya

**Keywords:** Ticks, Tick-borne pathogens, Chad, Djibouti, Ethiopia, Kenya, Tanzania, Uganda, Systematic review

## Abstract

**Background:**

Surveillance data documenting tick and tick-borne disease (TBD) prevalence is needed to develop risk assessments and implement control strategies. Despite extensive research in Africa, there is no standardized, comprehensive review.

**Methods:**

Here we tackle this knowledge gap, by producing a comprehensive review of research articles on ticks and TBD between 1901 and 2020 in Chad, Djibouti, Ethiopia, Kenya, Tanzania, and Uganda. Over 8356 English language articles were recovered. Our search strategy included 19 related MeSH terms. Articles were reviewed, and 331 met inclusion criteria. Articles containing mappable data were compiled into a standardized data schema, georeferenced, and uploaded to VectorMap.

**Results:**

Tick and pathogen matrixes were created, providing information on vector distributions and tick–pathogen associations within the six selected African countries.

**Conclusions:**

These results provide a digital, mappable database of current and historical tick and TBD distributions across six countries in Africa, which can inform specific risk modeling, determine surveillance gaps, and guide future surveillance priorities.

**Graphical Abstract:**

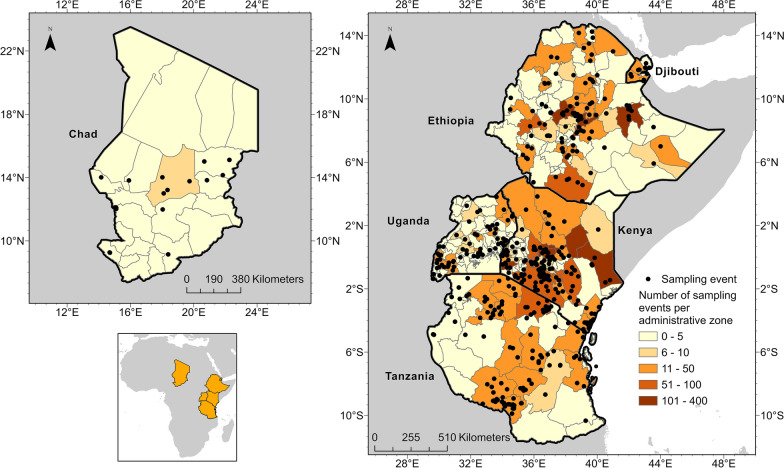

**Supplementary Information:**

The online version contains supplementary material available at 10.1186/s13071-023-06086-4.

## Background

Tick-borne disease (TBD) represents a growing threat to both human and animal health around the world. Increases in TBD burden over the last century may in part reflect improved surveillance and diagnostic capability. However, climate change and other ecological disturbances are also contributing to the displacement and/or expansion of tick habitats, which has further increased pathogen prevalence within tick populations [1, 2]. Global travel and international animal trade further facilitate the expanded distribution of certain tick species, as highlighted by the recent introduction and establishment of *Haemaphysalis longicornis* in the United States [3–5].

Sub-Saharan Africa is particularly vulnerable to the growing threat of ticks and TBD. An estimated 50% of the African continent’s livestock is found within the East Africa region, with livestock accounting for at least 20% of the agricultural gross domestic product (GDP) within Ethiopia, Kenya, and Uganda alone [6]. In Tanzania, around two-thirds of rural populations are reported to keep livestock [7]. Furthermore, roughly 1.5 million people maintain a pastoralist lifestyle within Kenya, Tanzania, and Uganda, creating ample opportunity for transboundary movement of ticks and TBD [8].

Previously reported TBDs in Eastern and Central sub-Saharan Africa include African tick bite fever (ATBF), Boutonneuse fever (BF), Crimean Congo hemorrhagic fever (CCHF), East Coast fever (ECF), Nairobi sheep disease (NSD), *Coxiella burnetii* (Q-fever), and  tick-borne relapsing fever (TBRF) [9]. While some of these diseases are characterized by febrile illness and skin rashes, others have more severe manifestations, such as CCHF, which is characterized by severe hemorrhagic disease, with reported fatality rates of up to 40% [10]. Diseases like East Coast fever, NSD, and Q-fever can also result in significant economic losses due to their effects on livestock health. In certain areas of Uganda, East Coast fever is responsible for as much as 50% of calf death within cattle production systems [11].

Despite the large number of tick collection studies that have been published over the last century and the known health risks and economic impacts of TBD, there is still no comprehensive resource for data on ticks or TBD presence in Africa. This lack of centralized knowledge undermines TBD mitigation and vector control efforts, leading to increased disease burden to humans and animals. To address this knowledge gap, our team conducted an expansive systematic literature review to describe the distribution of ticks and associated TBDs that have been reported from studies in Chad, Djibouti, Ethiopia, Kenya, Tanzania, and Uganda. Our primary objectives were to (1) identify peer-reviewed publications that contain high-quality, mappable tick collection data, (2) standardize data and georeference collection events, (3) submit data from systematic review to the VectorMap dashboard, a comprehensive country-specific database of tick species and pathogen distributions, and (4) identify surveillance gaps and other knowledge vacuums.

This open-access dataset will enhance various future analyses, as the data can be easily integrated with new collection data to model disease risk to humans and animals. By analyzing these data within a geographic information system (GIS) such as VectorMap, environmental and population data can be easily correlated with surveillance results, providing an opportunity to better characterize the risk profile of TBDs under current and future environmental and demographic conditions. Such information would prove highly beneficial to the regional economy and to veterinary and public health in sub-Saharan Africa.

### Methods

Nineteen Medical Subject Headings (MeSH) terms were used to search the PubMed, Scopus, Web of Science, and CABI VetMed databases. The details of all Boolean operators used across the databases are found in Table 1. Independent searches were conducted for all six countries in each of the selected databases. Searches targeted articles published from January 1901 through August 2020 and articles written in English. While our search strategy targeted articles written in English, we also translated any articles written in French identified during our search. The reference sections of each article meeting our inclusion criteria were also reviewed to accrue additional articles to evaluate that were not captured from the initial search results; these additional articles were reviewed in the same manner as those returned during database searches.

**Table 1:** Search terms used in the four search engines used for systematic review

PubMed	Scopus, Web of Science, CABI
(“Tick-Borne Diseases”[MeSH] OR	(“Tick-Borne Diseases” OR
“Rickettsia”[MeSH] OR	“Rickettsia” OR
“Anaplasmataceae”[MeSH] OR	“Anaplasmataceae” OR
“Borrelia”[MeSH] OR	“Borrelia” OR
“Babesia”[MeSH] OR	“Babesia” OR
“tick-borne zoonosis” OR	“tick-borne zoonosis” OR
“tick-borne zoonotic disease” OR	“tick-borne zoonotic disease” OR
“Seroepidemiologic Studies”[MeSH] OR	“Seroepidemiologic Studies” OR
“Hemorrhagic Fever virus, Crimean-Congo”[MeSH] OR	“Crimean-Congo Hemorrhagic Fever virus” OR
“Ticks”[MeSH] OR	“Ticks” OR
“*Amblyomma*” OR	“*Amblyomma*” OR
“*Dermacentor*” OR	“*Dermacentor*” OR
“*Haemaphysalis*” OR	“*Haemaphysalis*” OR
“*Hyalomma*” OR	“*Hyalomma*” OR
“*Ixodes*” OR	“*Ixodes*” OR
“*Margaropus*” OR	“*Margaropus*” OR
“*Rhipicephalus*” OR	“*Rhipicephalus*” OR
“*Ornithodoros*” OR	“*Ornithodoros*” OR
“*Argas*”)	“*Argas*”)
AND	AND
(*country*)	(*country*)

### Eligibility criteria

To determine whether an article should be included, the following exclusion criteria were used: studies conducted outside of geographical targets or with insufficient geographical data​, laboratory studies (e.g., vector competency, insectaries),​ and review articles (note: cited references were reviewed for original research). The inclusion criteria of articles were as follows: original research studies on tick species in the target countries of Central and East Africa including all life stages​, original research articles reporting TBD prevalence in humans/animals,​ and studies that included mappable collection data (e.g., sufficient geographical data). Included articles were combined and broken down by country (Additional file 3: Table S1).

Our team held weekly meetings to discuss questionable articles and to ensure that scrutiny was applied evenly when determining which articles met the set criteria. Articles were reviewed and tracked using guidance from the Preferred Reporting Items for Systematic Reviews and Meta-Analyses (PRISMA) checklist (Additional file 1: Figure S1). All articles that met our inclusion criteria were original research studies reporting results of tick collections and/or screening of TBDs in vectors or hosts. Included studies also reported sufficient information to determine the geographical origin of the collection events with measurable accuracy and precision. Our exclusion criteria identified non-peer-reviewed articles, articles reporting previously published results (reviews), or articles reporting ambiguous or dubious collection site descriptions. Any articles meeting our exclusion criteria were eliminated from our review.

### Data management

The process of assessing articles for inclusion eligibility was documented using separate MS Excel spreadsheets for each country. All articles captured using the Boolean search terms across all four databases were compiled into a single document for each country. The lists of returned articles then underwent the criteria stages outlined in PRISMA, with each stage documented as a separate sheet. First, duplicate articles were removed to provide a single list of unique articles returned from all four databases. After duplicate removal, articles were reviewed first by title and then by abstract to assess for article relevance. Any articles that did not meet the inclusion criteria were removed and documented during these steps. The remaining articles underwent a full review, and any that met the a priori inclusion criteria were used for data mining, as described below. A reference library containing all articles meeting our inclusion criteria was built and maintained using Zotero citation management software.

### Data mining

Data mining of eligible articles was conducted using a customized data schema established by the VectorMap project, which captures 93 fields of information, and a spreadsheet collection form, prompting different pieces of information to be extracted for each collection event entry. The extensive design ensured that as much information as possible was collected verbatim from the article as well as offering sections for unique aspects of the collection events. Data collected included tick/host identification methods and taxonomy, collection event locality descriptions, elevation and geographical coordinates, individual tick count, sex and life stage, collection methods, collection event habitat, and pathogen detection methods and results. Concerning the collection event locality, the most specific location information made available for each tick collection event was recorded. New entries were made for each unique collection event reported within the articles, separating by collection method and collection date whenever possible. Separate data entries were also created for different tick species collected during the same collection event. The extracted dataset for this work, including all references, Global Positioning System (GPS) points, pathogen detection results, and details on individual manuscripts, can be found within Additional file 2: Dataset S1.

### Georeferencing

Geographical data associated with each collection event entry were divided into two categories: those reporting geo-coordinates and those reporting named places only. For locations with geo-coordinates, we converted the latitude and longitude values to decimal degrees and calculated a spatial uncertainty measurement using the point-radius method [12, 13]. Locations described solely as named places were georeferenced using keyword searches retrieved from an online gazetteer (GeoNames.org). The spatial uncertainty of named places was calculated by measuring the distance between the locality’s centroid provided by the gazetteer to the farthest edge of the administrative border of that geographical entity [13]. If the collection event locality information was unclear, georeferencing was performed using the next most specific location information (e.g., province- or country-level information). Geographical data visualizations were generated using ArcGIS Pro 2.7.3 (Esri) and country shapefiles from the Database of Global Administrative Areas (GADM, version 4.0, https://gadm.org/).

## Results

**Fig. 1:**
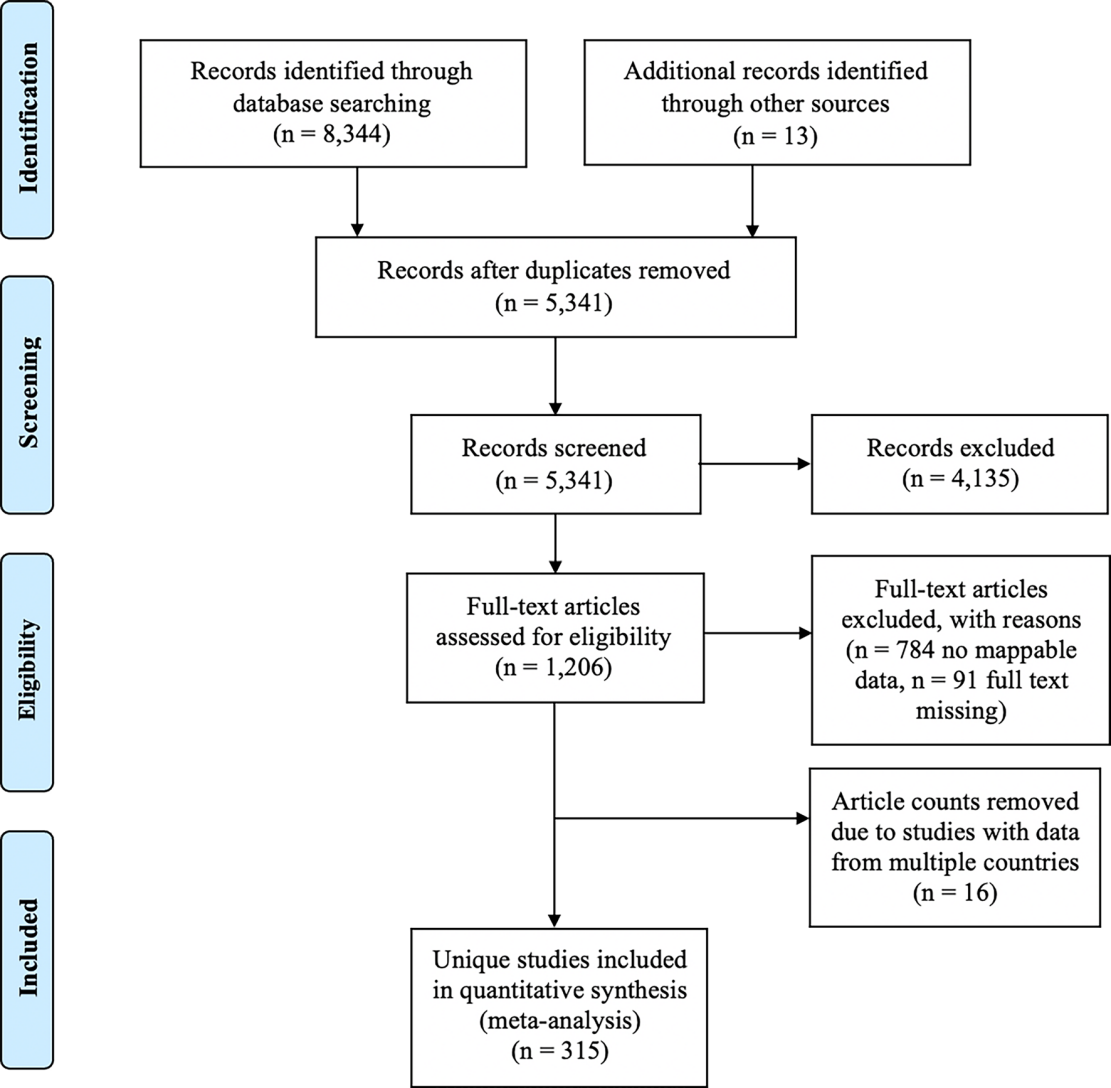
PRISMA inclusion/exclusion flowchart for all countries

**Fig. 2:**
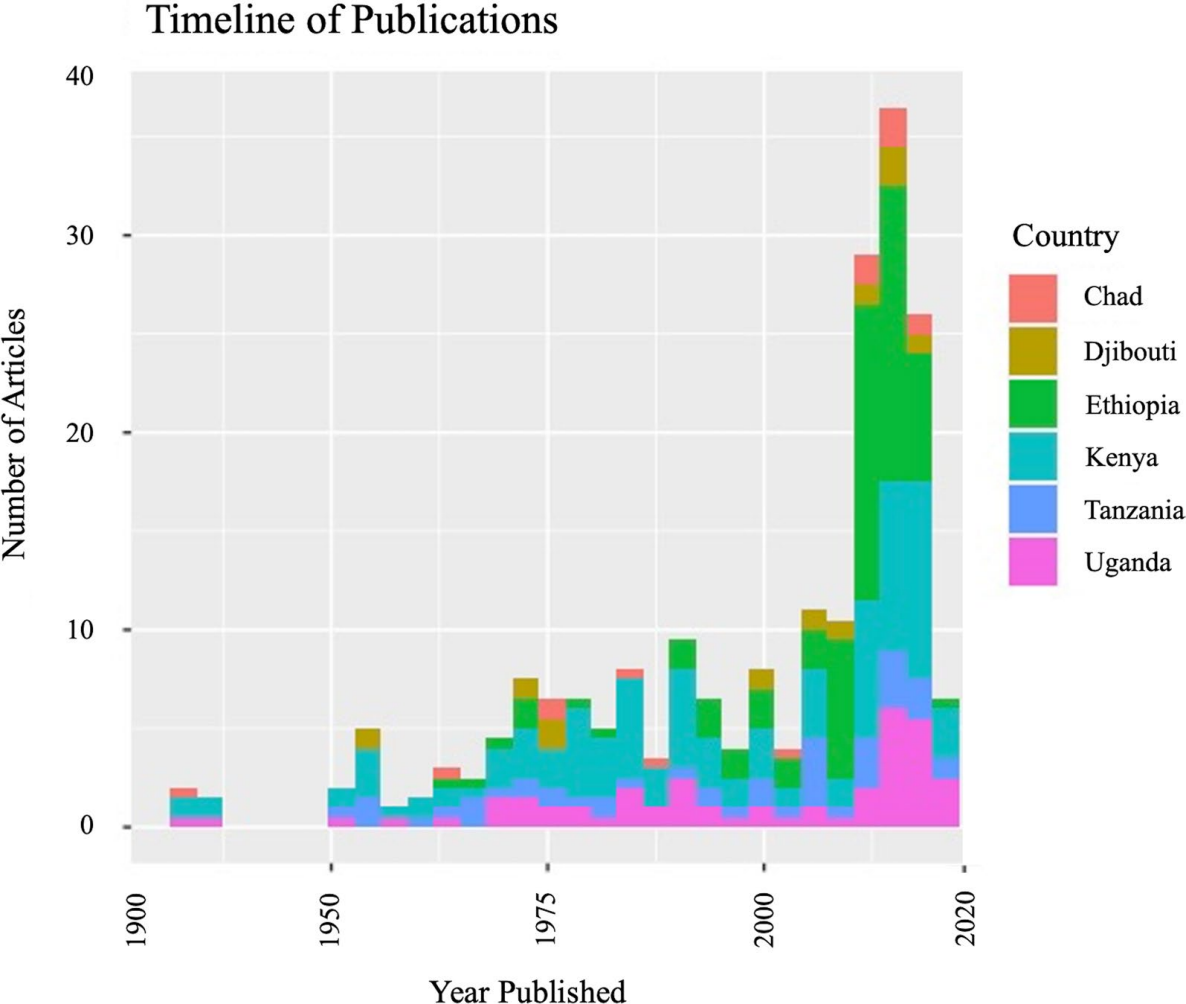
Timeline of publications by country

A total of 8357 articles, reporting data from our six target countries, were initially captured using the four search engines (CABI = 3176, PubMed = 2172, Scopus = 1754, and Web of Science = 1242), of which 315 articles met the final inclusion criteria and underwent data extraction and georeferencing. The stepwise results of the inclusion/exclusion process for the entire systematic review can be found in Fig. 1. A breakdown of the inclusion/exclusion process by country can be found in Table 2. A graphical timeline summary of all publications that met the final inclusion criteria is presented in Fig. 2. A total of 91 articles identified during our search were not able to be located after exhausting the interlibrary loan systems at each of our affiliated institutions. A full list of work contributing to this dataset can be found in Supplemental Table 1. There were 315 articles which met inclusion criteria for data extraction from Chad [14–19], Djibouti [15, 20–26], Ethiopia [15, 27–138, 154, 156, 175, 176], Kenya [15, 138–258], Tanzania [141, 177, 243, 259–298], and Uganda [15, 26, 145, 156, 175, 299–326].

**Table 2:** Country breakdown of article inclusion at each criteria stage

Database	Chad	Djibouti	Ethiopia	Kenya	Tanzania	Uganda	Totals
CABI	38	61	406	1853	551	267	3176
Web of Science	32	10	186	668	205	141	1242
Scopus	37	27	476	549	391	274	1754
PubMed	37	27	450	835	454	369	2172
Additional from secondary sourcing	0	4	0	2	7	0	13
Total	144	129	1518	3907	1608	1051	**8357**
Removed duplicates	46	45	606	1316	606	397	3016
Removed based on title	34	64	327	1697	654	155	2931
Removed based on abstract	28	4	259	491	227	195	1204
Removed based on article	29	7	186	244	65	253	784
Adjusting for multicountry studies^a^	1	1	5	1	3	5	16
Inaccessible^b^	1	0	23	37	12	18	91
Total extracted	5	8	112	121	41	28	315

^a^12 final included articles focused on multiple countries

^b^The full articles were unrecoverable through databases, online searches, and interlibrary loan requests

### Study results

**Fig. 3:**
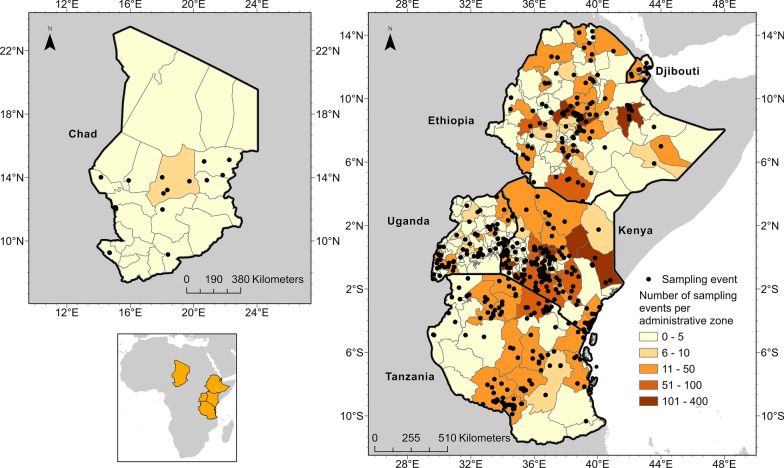
Map of tick collection events in Chad, Djibouti, Ethiopia, Kenya, Tanzania, and Uganda (1901–2020)

Across the six countries, collection records for six hard tick and two soft tick genera were captured. Additionally, the *Nuttalliella* ticks (Ixodoidae: Nuttalliellidae), which share morphological features of both hard and soft tick genera, were identified in reports from Tanzania [327]. Ticks of the genus *Rhipicephalus* were found to be the most diverse, with 43 species reported, while the *Dermacentor* and *Nuttalliella* genera were only mentioned once in the literature. *Amblyomma variegatum* was commonplace, with 414 unique collection event entries, followed by *Rhipicephalus evertsi evertsi* (*n* = 354) and *Rhipicephalus decoloratus* (*n* = 335). Among a total of 4305 unique collection entries, 3909 (90.8%) described ticks that were removed from an animal, which may lead to skewed reporting of tick species of veterinary importance when compared to tick diversity from environment sampling. A total of 10 different genera of bacterial pathogens, four different genera of protozoa, and 16 viruses were identified in ticks across all six countries. *Rickettsia* spp. was the most frequently reported bacterial genus overall, with 128 unique collection event entries reporting *Rickettsia africae.* Other commonly reported medically relevant bacteria included *Anaplasma bovis* (*n* = 56), *Anaplasma platys* (*n* = 77), *Ehrlichia canis* (*n* = 50), and *Ehrlichia ruminantium* (n = 89). The most frequently reported viruses were CCHF virus (CCHFV) (*n* = 15) and Dugbe virus (DUGV) (*n* = 19). Of the 825 unique collection event entries that included an associated pathogen, 85.2% of these described ticks which were collected from an animal. Maps displaying all unique collection sites are provided in Fig. 3, with a summary of tick species captured by country in Table 3.

**Table 3:** Comprehensive list of all tick species identified in the articles meeting inclusion criteria. Reported presence (+) in countries is indicated for each tick species

	Chad	Djibouti	Ethiopia	Kenya	Tanzania	Uganda
Africaniella	−	−	**−**	**−**	**−**	**−**
*A. transversale*	**−**	**−**	**−**	**−**	**+**	**−**
Amblyomma	−	−	**+**	**+**	**+**	**+**
*A. cohaerens*	−	**+**	**+**	**+**	**+**	**+**
* A. detritum*	−	**+**	−	−	−	−
* A. eburneum*	−	−	−	**+**	**+**	−
* A. exornatum*	−	−	−	−	**+**	−
*A. falsomarmoreum*	−	**+**	−	**+**	**+**	−
* A. gemma*	−	**+**	**+**	**+**	**+**	**+**
* A. hebraeum*	−	−	**+**	**+**	**+**	−
* A. latum*	−	−	−	**+**	**+**	−
* A. lepidum*	−	**+**	**+**	**+**	**+**	**+**
* A. marmoreum*	−	**+**	−	−	**+**	−
* A. nuttalli*	−	−	−	**+**	**+**	−
* A. paulopunctatum*	−	−	−	−	−	**+**
* A. personatum*	−	−	−	−	**+**	−
* A. rhinocerotis*	−	−	−	−	**+**	−
* A. sparsum*	−	−	−	**+**	**+**	−
*A. tholloni*	−	−	−	**+**	**+**	−
*A. variegatum*	**+**	**+**	**+**	**+**	**+**	**+**
Argas	−	−	−	−	**+**	−
*Ar. africolumbae*	−	−	−	**+**	**+**	−
*Ar. arboreus*	−	−	**+**	−	−	−
*Ar. brumpti*	−	−	−	**+**	**+**	−
*Ar. persicus*	−	−	**+**	**+**	**+**	−
*Ar. vespertilionis*	−	−	−	**+**	−	−
Carios	−	−	−	−	**-**	−
*C. erraticus*	−	−	−	**+**	−	**+**
*C. faini*	−	−	−	−	−	**+**
Dermacentor	−	−	−	−	**+**	−
*D. rhinocerinus*	−	−	−	**+**	**+**	−
Haemaphysalis	−	−	**+**	**+**	**+**	−
*Hae. aciculifer*	−	−	**+**	**+**	**+**	**+**
*Hae. houyi*	−	−	-	**+**	−	−
*Hae. leachi*	−	−	**+**	**+**	**+**	**+**
*Hae. muhsamae*	−	−	-	−	**+**	−
*Hae. paraleachi*	−	−	**+**	**+**	−	**+**
*Hae. parmata*	−	−	**+**	**+**	−	−
*Hae. princeps*	**+**	−	−	−	−	−
*Hae. punctaleachi*	−	−	−	−	−	**+**
*Hae. rugosa*	−	−	−	−	−	**+**
*Hae. spinulosa*	−	−	**+**	−	−	−
*Hae. subterra *	−	−	**+**	**+**	−	−
*Hae. walkerae*	−	−	−	**+**	−	−
Hyalomma	**+**	**+**	**+**	**+**	**+**	**+**
*Hy. aegyptium*	−	−	−	**+**	−	−
*Hy. albiparmatum*	−	−	**+**	**+**	**+**	−
*Hy. anatolicum*	−	**+**	**+**	−	−	−
*Hy. dromedarii*	−	+	**+**	**+**	−	−
*Hy. excavatum*	−	**+**	**+**	−	−	−
*Hy. impeltatum *	**+**	**+**	**+**	**+**	**+**	−
*Hy. impressum *	−	−	−	**+**	−	−
*Hy. lusitanicum*	**+**	−	−	−	−	−
*Hy. marginatum *	**+**	**+**	**+**	**+**	**+**	−
*Hy. punt*	−	−	**+**	−	−	−
*Hy. rufipes*	**+**	**+**	**+**	**+**	**+**	**+**
*Hy. scupense*	−	−	−	**+**	−	−
*Hy. somalicum*	−	−	**+**	−	−	−
*Hy. truncatum*	−	**+**	**+**	**+**	**+**	**+**
Ixodes	−	−	**+**	**+**	**+**	−
*I. alluaudi *	−	−	−	**+**	−	−
*I. cavipalpus*	−	−	−	**+**	**+**	−
*I. cumulatimpunctatus*	−	−	−	**+**	−	−
*I. latus*	−	−	−	−	**+**	−
*I. lewisi*	−	−	−	**+**	−	−
*I. muniensis*	−	−	−	**+**	−	−
*I. nairobiensis*	−	−	−	−	**+**	−
*I. pilosus *	−	−	−	**+**	−	−
*I. schillingsi*	−	−	−	−	**+**	−
*I. thomasae*	−	−	−	**+**	−	−
*I. ugandanus*	−	−	−	−	**+**	−
*I. walkerae*	−	−	−	**+**	−	−
Nuttalliella	−	−	−	−	**+**	−
*Nuttalliella namaqua*	−	−	−	−	**+**	−
Ornithodoros	**+**	−	**+**	−	**+**	−
*O. capensis*	−	−	**+**	−	**+**	−
*O. coniceps*	−	−	−	**+**	−	−
*O. graingeri*	−	−	−	**+**	−	−
*O. moubata*	−	−	−	**+**	**+**	**+**
*O. porcinus*	−	−	−	−	**+**	−
*O. savignyi*	−	**+**	−	−	−	−
*O. vansomereni*	−	−	−	**+**	−	−
Rhipicephalus	−	**+**	**+**	**+**	−	**+**
*Rh. afranicus*	−	−	−	−	−	**+**
*Rh. annulatus*	**+**	**+**	**+**	**+**	−	−
*Rh. appendiculatus *	−	−	−	**+**	**+**	**+**
*Rh. aquatilis*	−	−	−	−	−	**+**
*Rh. armatus*	−	−	−	**+**	−	−
*Rh. bergeoni*	−	−	**+**	−	−	−
*Rh. bursa*	−	−	−	**+**	−	−
*Rh. camicasi*	−	**+**	**+**	**+**	−	−
*Rh. capensis*	−	−	−	**+**	−	−
*Rh. compositus*	−	−	−	**+**	**+**	−
*Rh. decoloratus*	**+**	**+**	**+**	**+**	**+**	**+**
*Rh. distinctus *	−	−	−	**+**	**+**	−
*Rh. evertsi evertsi*	−	**+**	**+**	**+**	**+**	**+**
*Rh. guilhoni*	**+**	**+**	**+**	−	−	−
*Rh. humeralis*	−	−	−	**+**	**+**	−
*Rh. Hurti*	−	−	−	**+**	**+**	**+**
*Rh. interventus*	−	−	−	−	**+**	−
*Rh. jeanneli*	−	−	−	**+**	**+**	−
*Rh. kochi*	−	−	−	**+**	−	−
*Rh. longicoxatus*	−	**+**	**+**	−	**+**	−
*Rh. longus *	−	−	**+**	−	**+**	**+**
*Rh. lunulatus*	−	−	**+**	−	−	−
*Rh. maculatus*	−	−	−	**+**	**+**	−
*Rh. masseyi*	−	−	−	−	**+**	−
*Rh. microplus*	−	−	−	**+**	**+**	**+**
*Rh. muehlensi*	−	−	−	**+**	**+**	−
*Rh. muhsamae*	−	−	**+**	−	**+**	**+**
*Rh. planus*	−	−	−	**+**	−	−
*Rh. pravus*	−	**+**	**+**	**+**	**+**	**+**
*Rh. praetextatus*	−	**+**	**+**	**+**	−	**+**
*Rh. pulchellus*	−	**+**	**+**	**+**	**+**	**+**
*Rh. punctatus *	−	−	−	−	**+**	−
*Rh. sanguineus s.l.*	**+**	**+**	**+**	**+**	**+**	**+**
*Rh. sculptus*	−	−	−	−	**+**	−
*Rh. simpsoni*	−	−	−	**+**	−	−
*Rh. simus*	−	−	−	**+**	**+**	**+**
*Rh. sulcatus*	**+**	−	**+**	−	**+**	−
*Rh. supertritus*	−	−	−	**+**	**+**	−
*Rh. tricuspis*	−	−	−	−	**+**	**+**
*Rh. turanicus*	**+**	−	−	−	−	**+**

#### Chad

**Fig. 4:**
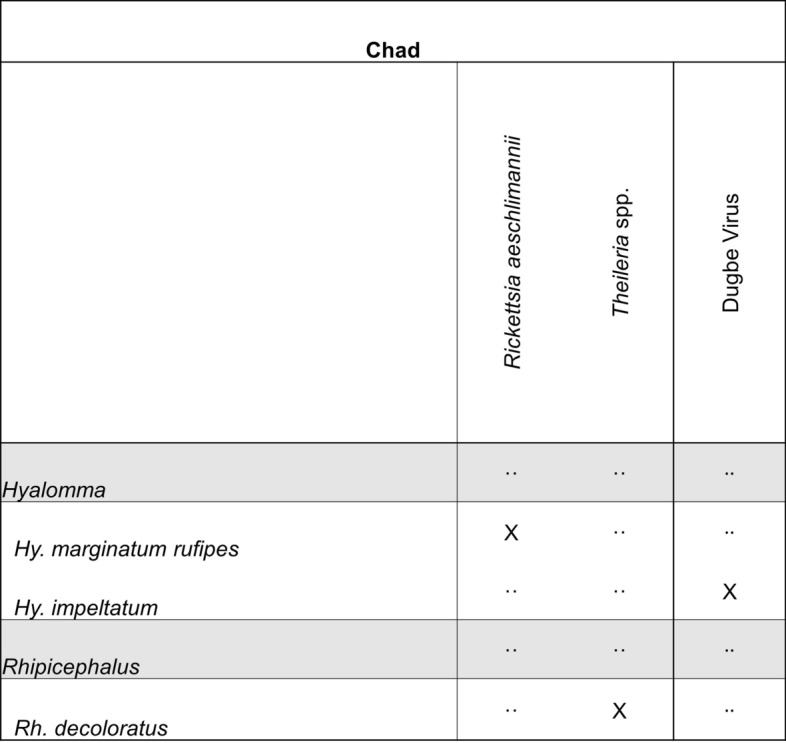
Tick pathogen profile—Chad

A total of 144 unique papers were captured for Chad from the four literature databases, of which 36 articles met the inclusion criteria for a full review, and only six articles reported collection data that met our quality inclusion criteria. These articles produced surveillance records for five unique genera: *Amblyomma*, *Haemaphysalis*, *Hyalomma*, *Ornithodoros*, and *Rhipicephalus*. Twelve unique tick species were identified from these reports, including six *Rhipicephalus* species. *Rhipicephalus guilhoni* had the greatest number of unique collection event entries within Chad, followed by unspecified *Ornithodoros* species. Genetic material from DUGV was detected within *Hyalomma impeltatum* ticks, alluding to the presence of DUGV in Chad [328]. Other microbial species identified within reports from Chad included *Rickettsia aeschlimannii* detected in *Hyalomma rufipes* collected from camels and *Theileria* spp. from *Rh. decoloratus* collected from Chadian saddle horses (Fig. 4). Most collection events occurred near N’Djamena, while many other collection events occurred within the Batha Ouaddai and Wadi Fira regions.

#### Djibouti

**Fig. 5:**
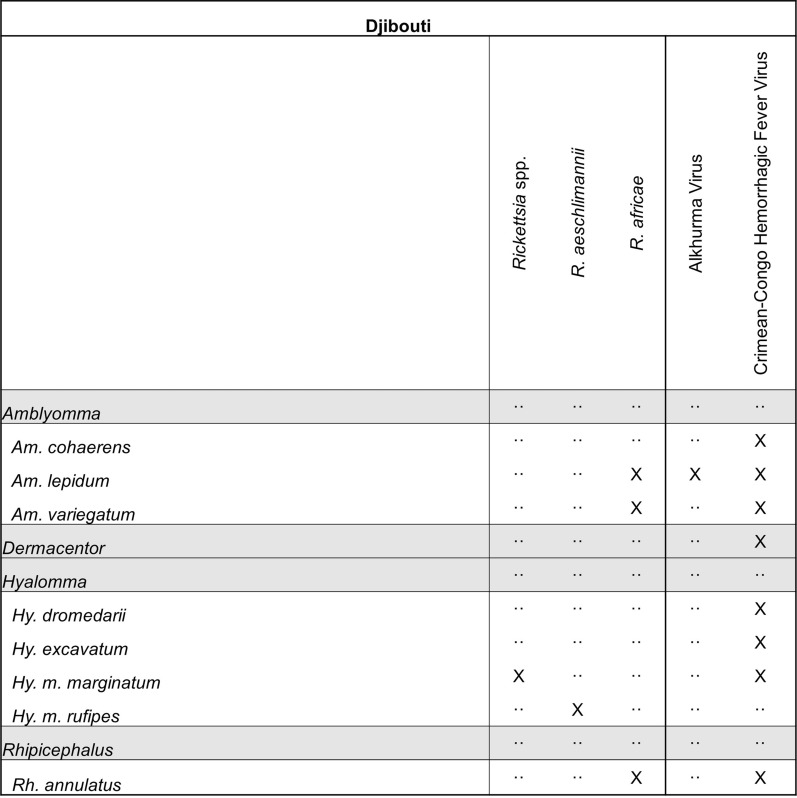
Tick pathogen profile—Djibouti

A total of 129 unique titles were initially identified from Djibouti during our review. Of those, eight articles met our inclusion criteria and reported quality collection data. In total, 23 species of ticks spanning five different genera were reported. Ticks of the genera *Hyalomma* and *Rhipicephalus* had the greatest number of unique collection events. *Rhipicephalus camicasi* and *Rhipicephalus sanguineus* were the most common species reported. Pathogens detected from Djibouti include Alkhurma virus (AHF), CCHFV, *R. aeschlimannii*, and *R. africae* (Fig. 5). Most ticks reported were removed from animals (97/106 data mining event entries), including all instances of tick specimens or pools screened for pathogens. The most common locations reporting tick collection events in Djibouti were Dikhil, Tadjourah, and Ali Sabieh regions and the capital Djibouti City.

#### Ethiopia

**Fig. 6:**
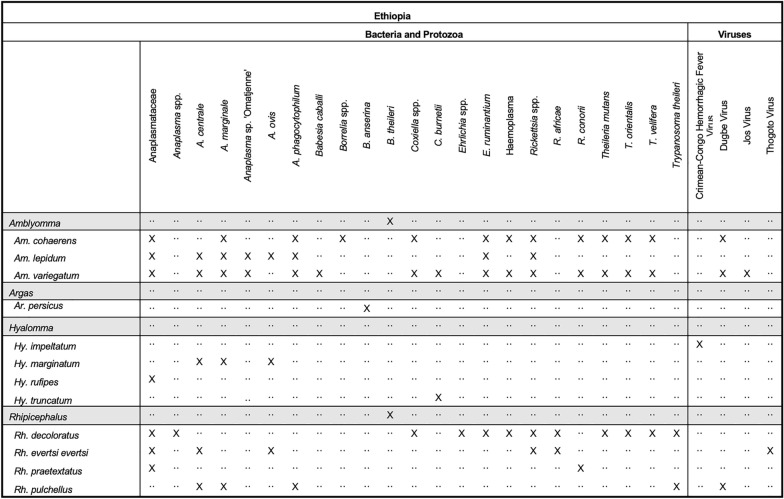
Tick pathogen profile—Ethiopia

A total of 113 articles reporting tick and TBD collections in Ethiopia met our inclusion criteria. A total of 44 species representing seven genera of ticks were identified including five hard tick genera (*Amblyomma*, *Haemaphysalis*, *Hyalomma*, *Ixodes*, and *Rhipicephalus*) and two soft tick genera (*Argas* and *Ornithodoros*). Sixteen species of *Rhipicephalus* ticks were reported, followed by nine different species/subspecies of *Hyalomma* ticks. The most frequently reported tick species was *Am. variegatum*, with approximately 73.3% of articles detecting this species. *Rhipicephalus decoloratus* and *Rh. evertsi evertsi* were reported in 63.8% and 56.9% of studies, respectively. A total of 26 microbial organisms were reported from Ethiopia, including four viruses: CCHFV, DUGV, Jos (JOSV), and Thogoto (THOV) viruses. Additional microbial organisms include the medically important genera *Anaplasma*, *Borrelia*, *Coxiella*, *Ehrlichia*, *Rickettsia*, *Babesia*, *Theileria*, and *Trypanosoma* (Fig. 6). *Amblyomma* and *Rhipicephalus* ticks were the most frequently reported genera with associated pathogens. Some 97.8% of tick collections were from animals, most of which were livestock species such as cattle, sheep, goats, and camels. The most commonly surveyed areas include the regions surrounding Addis Ababa (North, East and West Shewa districts) and Dire Dawa (East Hararghe district).

#### Kenya

Data were extracted from 121 articles reporting tick surveillance in Kenya that met our inclusion criteria. Over 60 unique tick species were compiled during data mining, representing eight different genera: six hard ticks (*Amblyomma*, *Dermacentor*, *Haemaphysalis*, *Hyalomma*, *Ixodes*, *Rhipicephalus*) and three soft ticks (*Argas, Carios, Ornithodoros*). The tick species with the largest number of unique data extraction entries were *Rh. appendiculatus*, *Rh. pulchellus*, *Rh. evertsi evertsi*, and *Am. variegatum*. Of these, 88.6% of georeferenced tick entries were removed from domestic or wild animals. Of note, 36.7% of data extraction event entries reported a microbial organism associated with the tick. Bacteria, viruses, and protozoa were observed within the collection entries compiled from these articles, including over 400 instances of *Rickettsia*.

**Fig. 7:**
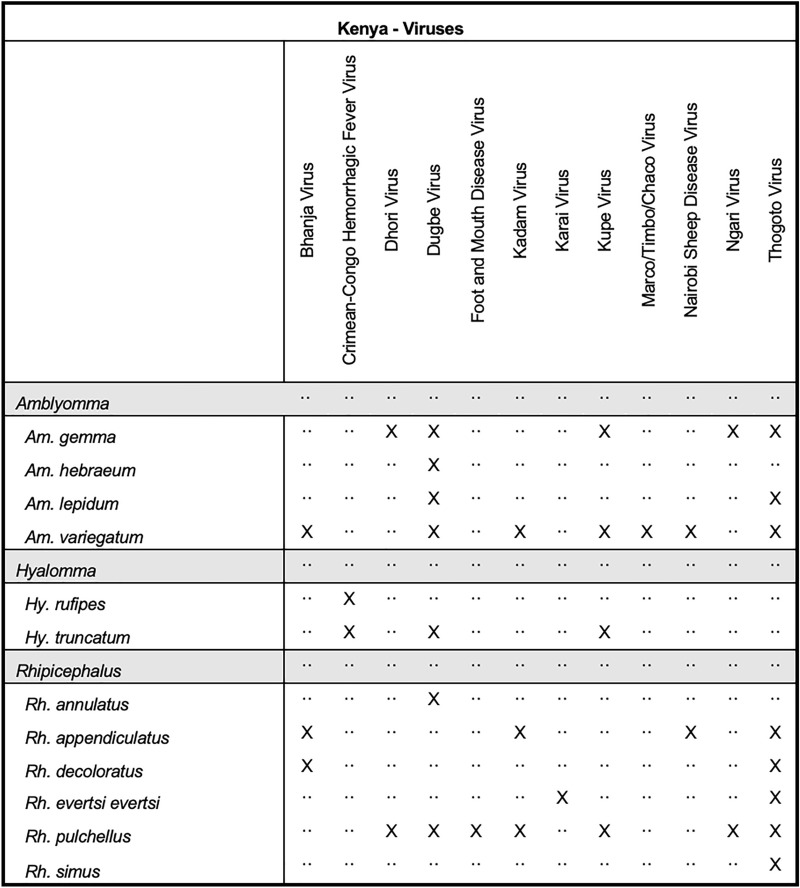
Tick pathogen profile—Kenya (viruses)

**Fig. 8:**
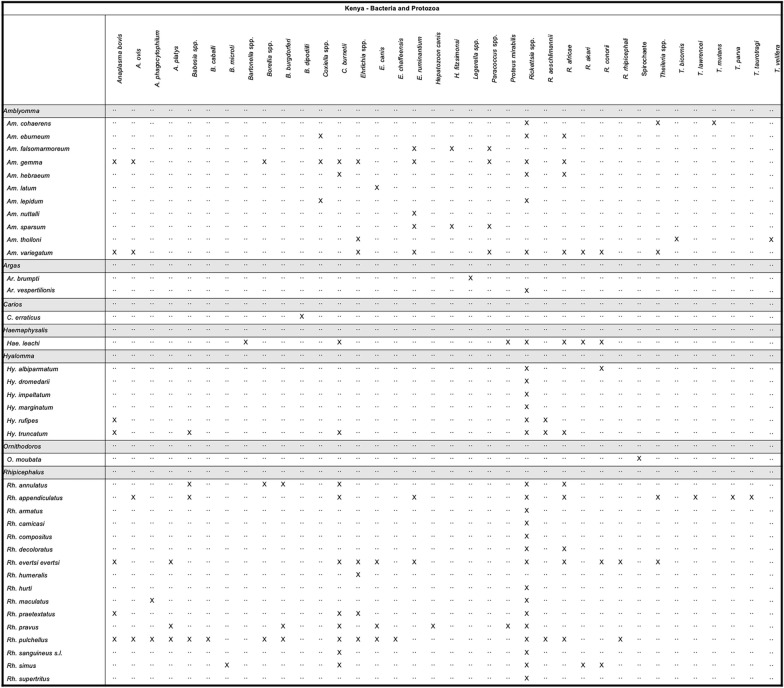
Tick pathogen profile—Kenya (bacteria and protozoa)

Other microbial species reported from collected ticks include viruses such as Bhanja (BHAV), CCHFV, Dhori (DHOV), DUGV, Kadam (KADV), Kupe (KUPV), NSD, Ngari (NRIV), and THOV viruses, as well as *Anaplasma* spp., *Borrelia* spp., *Coxiella* spp., *Ehrlichia* spp., *Babesia* spp., *Hepatozoon* spp., and *Theileria* spp., (Figs. 7, 8). The tick species most frequently reported with an associated microbial organism included *Rh. appendiculatus*, *Rh. pulchellus*, *Rh. evertsi evertsi*, and* Am. variegatum*. Some 84.9% (558/657) of ticks reported with associated pathogens were removed from animals. Areas of Kenya that were more frequently surveyed include the area surrounding Nairobi, Garissa, Isiolo, Samburu, Laikipia, and Homa Bay counties.

#### Tanzania

**Fig. 9:**
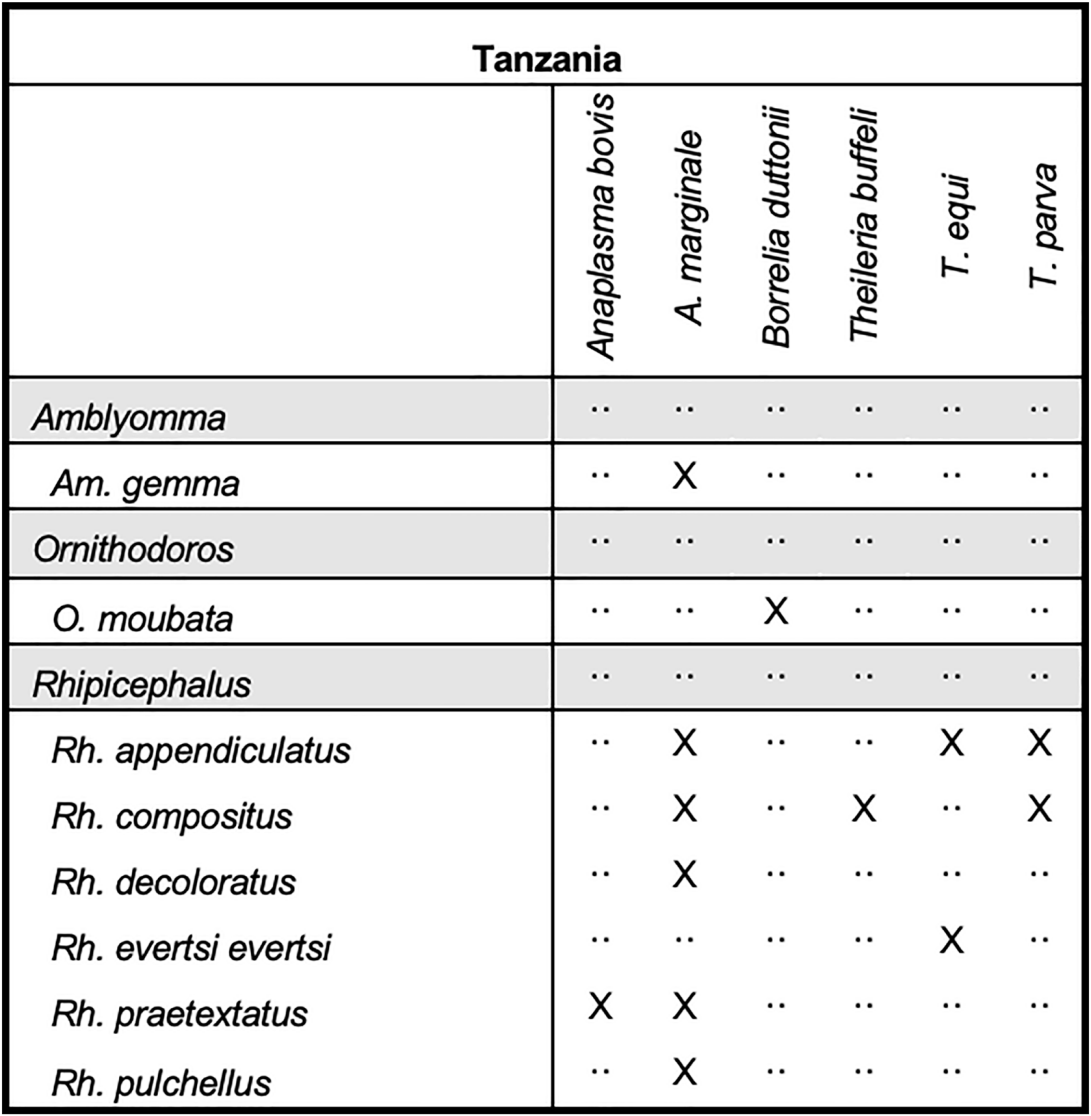
Tick pathogen profile—Tanzania

A total of 43 articles reporting tick surveillance from Tanzania met our inclusion criteria during this review. The articles were published between 1950 and 2020, with most entries published in the 1970s. Across all included articles, 68 species spanning nine tick genera were reported, including the only record of *Nuttalliella* spp. compiled during this review. A total of 24 species were identified within the genus *Rhipicephalus*, with *Rh. appendiculatus* reporting the highest number of unique collection event entries (*n* = 78), followed by *Am. variegatum* (*n* = 48) and *Rh. evertsi evertsi* (*n* = 44). Over 90% of the unique collection event entries described ticks that were collected from wild or domestic animals. Microbial species that were detected within collection events included members from the following genera: *Borrelia*, *Anaplasma,* and *Theileria* (Fig. 9). In contrast to the other countries included in this review, only 7.7% of pathogen-positive ticks were collected from animals. The collections occurred primarily in the northern, central, eastern, and western regions, with the most collections occurring within the Arusha and Singida regions.

#### Uganda

**Fig. 10:**
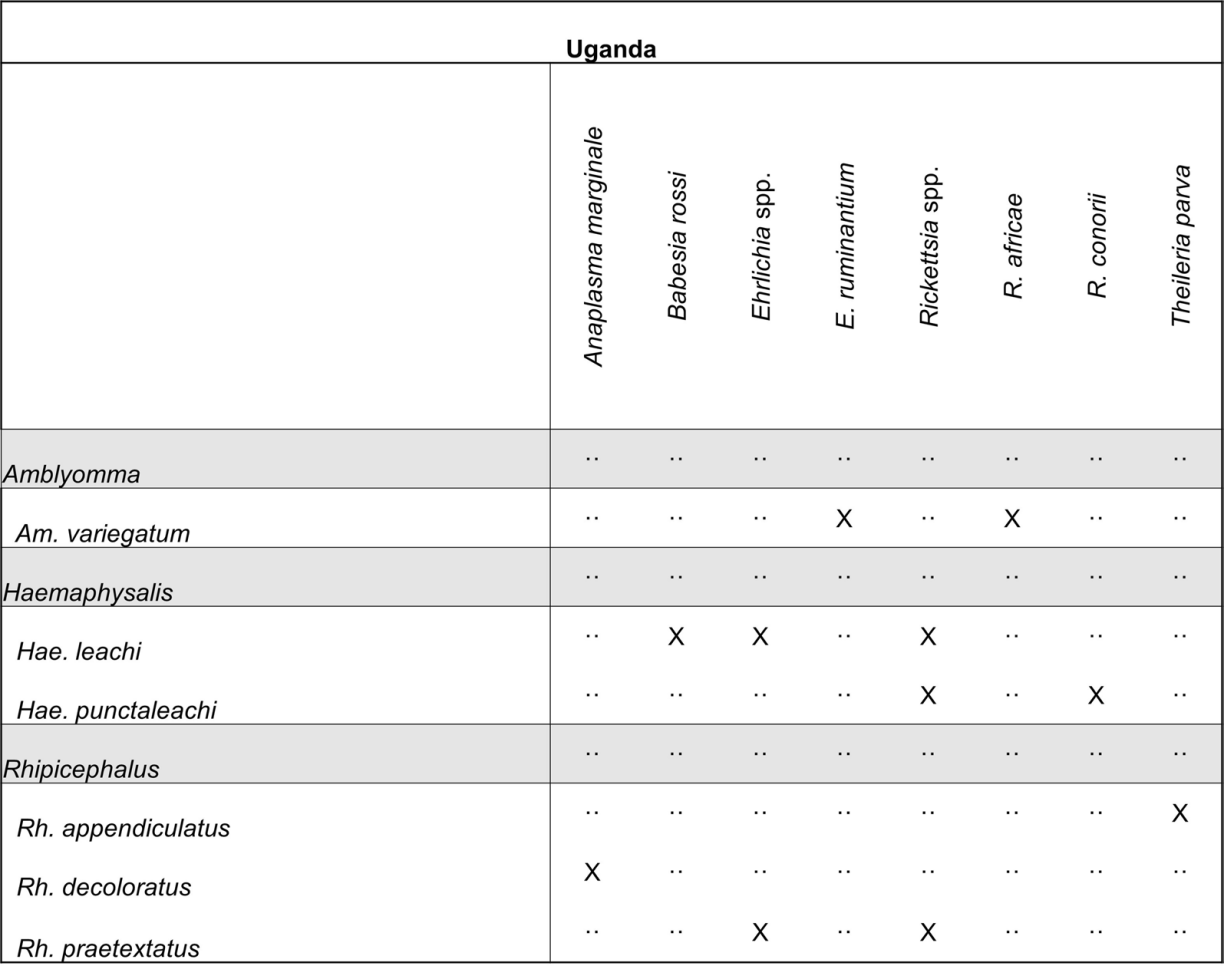
Tick pathogen profile—Uganda

A total of 28 unique articles from Uganda met our inclusion criteria. Included articles were published between 1952 and 2020, with an increase in publications occurring between 2015 and 2020. Five articles were published in 2020, which represents the most articles in a single year for Uganda. Ticks from 35 species representing five genera were collected across all articles, including *Amblyomma*, *Haemaphysalis*, *Hyalomma,* and *Rhipicephalus* hard ticks, and soft ticks of the genera *Carios* and *Ornithodoros*. The largest number of species identified within a single genus was *Rhipicephalus*, with 15 species reported from Uganda. *Amblyomma variegatum* (*n* = 20) was the most frequently reported tick species, followed by *Rh. appendiculatus* (*n* = 18) and *Rh. decoloratus* (*n* = 15). Microbial groups reported from collected ticks include species of *Anaplasma*, *Ehrlichia*, *Rickettsia*, *Babesia*, and *Theileria* (Fig. 10). In total, 92.4% of tick entries were those collected from animals, with cattle being the most common. All tick collection event entries with an associated pathogen group describe a tick collected off either a cow or dog. Most collection events occurred in the southwest and northeast regions of Uganda, as well as around the country’s capital Kampala. A final list of all pathogens detected within ticks from this literature review can be found in Table 4.

**Table 4:** Comprehensive list of all pathogens identified in ticks from this study

Bacteria	Protozoa
*Anaplasma* spp.	*Babesia* spp.
*A. bovis*	*B. caballi*
*A. centrale*	*B. microti*
*A. marginale*	*B. rossi*
*Anaplasma* sp. “Omatjenne”	*Hepatozoon* spp.
*A. ovis*	*H. canis*
*A. phagocytophilum*	*H. fitzsimonsi*
*A. platys*	*Legerella* spp.
*Anaplasmataceae*	*Theileria* spp.
*Bartonella* spp.	*T. bicornis*
*Borrelia* spp.	*T. buffeli*
*B. anserina*	*T. equi*
*B. burgdorferi*	*T. lawrencei*
*B. dipodilli*	*T. mutans*
*B. duttonii*	*T. orientalis*
*B. theileri*	*T. parva*
*Coxiella* spp.	*T. taurotragi*
*C. burnetii*	*T. velifera*
*Ehrlichia* spp.	*Trypanosoma theileri*
*E. canis*	Viruses
*E. chaffeensis*	Alkhurma virus (AHF)
*E. ruminantium*	Bhanja virus (BHAV)
Haemoplasma	Crimean-Congo hemorrhagic fever virus (CCHFV)
*Paracoccus* spp.	Dhori virus (DHOV)
*Proteus mirabilis*	Dugbe virus (DUGV)
*Rickettsia* spp.	Foot-and-mouth disease virus (FMDV)
*R. aeschlimannii*	Jos virus (JOSV)
*R. africae*	Kadam virus (KADV)
*R. akari*	Karai virus (KSIV)
*R. conorii*	Kupe virus (KUPV)
*R. rhipicephali*	Marco/Timbo/Chaco virus (MCOV, TIMV, CHOV)
Spirochaete	Nairobi sheep disease (NSD) virus
	Ngari virus (NRIV)
	Thogoto virus (THOV)

## Discussion

The ability for public health stakeholders to create policies supporting TBD prevention and control is dependent on a thorough understanding of the epidemiology and distribution of tick species and detailed information regarding the clinically relevant pathogens they carry. While gaps remain, such information is available but hard to access, being scattered among numerous published articles. Without incorporation into a single, standardized database, it is difficult to integrate historical baseline data with current surveillance efforts. We sought to address this critical knowledge gap by integrating over 100 years' worth of literature into a singular, standardized database that continues to grow as new surveillance data are generated. Information collected from our review can be used to predict tick and pathogen distribution and create pathogen risk profiles, which can inform appropriate preventative measures for medical and veterinary health care professionals while also identifying gaps where additional surveillance is needed.

By systematically collating and georeferencing data from the past century on TBDs, we have developed a single dataset that can be used by both researchers and policymakers in Africa. While this database will prove useful for future risk assessment analyses, it also identifies major gaps in tick and TBD surveillance within the region, and thus can be used to guide future surveillance studies. Most data reported in the literature document collections of ticks from animals. Although this is a valuable source of data, these studies do not capture the full range of tick species that threaten human and animal health or the ecological dynamics of pathogens with their vectors. Environmental sampling is needed to further understand the dynamic relationships between ticks, their hosts, TBD, and the environment, and future studies should target analyzing the pathogens present within ticks from the environment and the blood meals they may have obtained from feeding on wild animal hosts. Our database clearly identifies the need for enhanced environmental sampling within all countries included in the study. Overall, only 7.9% of data entries represented tick collection events from the environment, with country-level proportions ranging from 2.3% (Ethiopia) to 30.4% (Chad).

Other gaps in surveillance relate to tick identification and the reporting of tick life stage. Only 2008 tick collection event entries report what life stage of each species was captured, which undermines identification confidence for collection events that relied solely on morphological identification methods. It is also important to note the numerous taxonomic changes that have impacted the species names reported in the literature over the last century. While verbatim species names were updated to reflect current valid names, the component taxa within species complexes (e.g., *Rh. sanguineus* sensu lato) are in flux and distributions are likely to change [329]. Data presented here will be invaluable in determining historical species ranges, informing forthcoming taxonomic revisions. Given the temporal nature of this review, these data may also provide insights into the impact of land use and landscape change, global warming, and epidemiological shifts in disease presence, especially within the last two decades.

### Chad

This review highlighted the limited understanding of tick distributions in Chad. Most collection events reported in the literature were opportunistic tick surveys conducted on domestic animals, livestock, and wildlife, with very few collection events coming from vegetation and/or animal burrows. Of the ticks screened for pathogens, DUGV, *R. aeschlimannii*, and an unspecified *Theileria* spp. were detected. Equine piroplasmosis (EP), caused by *Babesia caballi* or *Theileria equi*, has been associated with up to a 50% mortality rate, although in endemic settings the mortality may be lower. Given the pathogenicity of EP agents, they represent a major threat to communities in Chad, as Dongola horses play a significant role in everyday operations for both the Chadian National Guard and nomadic community members.

### Djibouti

Like Chad, the results of the literature review demonstrate a limited characterization of the ticks found within Djibouti. Over 90% of collection events reported described ticks removed from animals. This indicates a major gap in the characterization of ticks within Djibouti, as studies detailing local environmental tick sampling are sparse. Pathogens detected within the sampled ticks included CCHFV in *Amblyomma* spp., *Dermacentor* spp., *Hyalomma* spp., and *Rhipicephalus* spp. ticks, AHF in *Amblyomma lepidum*, *R. africae* in *Amblyomma* spp. and *Rhipicephalus* spp., and *R. aeschlimannii* in *Hy. rufipes*. Both AHF and CCHFV have been reported to cause non-specific flu-like symptoms that can develop hemorrhagic manifestations, with case fatality rates over 15% [330]. Of note, several collection event entries documenting ticks collected from cattle at slaughterhouses within Djibouti were captured, and in some instances these cattle had been brought into Djibouti from neighboring countries. While sampling at slaughterhouses represents a convenient way to collect ticks, complications arise as to whether any identified ticks or TBD represent species endemic to Djibouti or endemic to the cattle’s country of origin. Given the sustained movement of both humans and animals from Africa to the Middle East through Djibouti, there is enhanced risk for the import or establishment of non-endemic TBDs within the country.

### Ethiopia

Numerous pathogens were detected in multiple species collected in Ethiopia. Agents detected included CCHFV, DUGV, JOSV, THOV, *Anaplasma centrale*, *Anaplasma marginale*, *Anaplasma ovis*, *Anaplasma phagocytophilum*, *Borrelia anserina*, *Borrelia theileri*, *Coxiella burnetii*, *E. ruminantium*, *R. africae*, *Rickettsia conorii*, *B. caballi*, *Theileria orientalis*, *Theileria mutans*, *Theileria velifera*, and *Trypanosoma theileri*. The presence of microbes from the *Anaplasmataceae* family and *Borrelia* species warrants concern regarding the well-being of livestock and the potential economic impact on pastoralists as it relates to the organisms responsible for human and animal anaplasmosis and ehrlichiosis [331]. *Anaplasma phagocytophilum* causes multiple diseases in different hosts: granulocytic anaplasmosis, ruminant tick-borne fever, and human granulocytic anaplasmosis (HGA) [332]. Humans with HGA experience symptoms that are like animal anaplasmosis, including anemia, hepatic injury, septic shock, and acute respiratory distress syndrome (ARDS) [333]. *Borrelia* spp. known to cause disease in domestic animals were also reported, including *B. anserina,* the causative agent of avian spirochetosis, which is characterized by high mortality in commercial bird species, and *B. theileri*, which is known to cause bovine borreliosis, a mild disease characterized by anemia and fever [334, 335] . *Rickettsia conorii* causes Mediterranean spotted fever, which typically presents as spotted fever, although complications such as vasculitis and multiple organ failure have also been reported [336, 337]. In addition, infections with certain *Theileria* spp. such as *T. annulata* and *T. parva* can cause anemia in cattle, impacting milk and meat production. One group estimated that the annual economic loss resulting from *T. parva* infections alone may cause losses as high as $168 million across regions of eastern, central, and southern Africa [338]. The detection of these numerous TBDs demonstrates a major threat to the people of Ethiopia. The collection records help establish baseline data. However, there is still a need for further surveillance in numerous regions in order to fully characterize the presence of endemic tick species and TBDs of Ethiopia.

### Kenya

Data for tick and TBD research conducted within Kenya was abundant compared to other countries targeted during this review. From these studies, 167 unique georeferenced collection sites and 1787 collection event entries were documented. Some 107 animal species were sampled, including livestock, domestic animals, large carnivores, herbivores, rodents, and reptiles. Of the six countries reviewed, Kenya had the highest number of publications reporting data on ticks and the pathogens they carry, with 68 documented tick species from eight genera (*Amblyomma*, *Argas*, *Dermacentor*, *Haemaphysalis*, *Hyalomma*, *Ixodes*, *Ornithodoros*, and *Rhipicephalus*). The genera with the highest number of unique species reported were *Rhipicephalus* and *Amblyomma*.

Within the collected data, etiological agents detected included CCHFV, DUGV, DHOV, BHAV, Karai virus (KSIV), KADV, NSD virus, NRIV, THOV, *A. bovis*, *A. ovis*, *A. phagocytophilum*, *A. platys*, *Bartonella* spp., *Borrelia burgdorferi*, *Borrelia dipodilli*, *C. burnetii*, *E. canis*, *Ehrlichia chaffeensis*, *E. ruminantium*, *Paracoccus* spp., *Proteus mirabilis*, *R. aeschlimannii*, *R. africae*, *Rickettsia akari*, *R. conorii*, *Rickettsia rhipicephali*, *B. caballi*, *Babesia microti*, *Hepatozoon canis*, *Hepatozoon fitzsimonsi*, *T. parva*, *Theileria taurotragi*, and *T. velifera*. *Rickettsia africae* was the most frequently detected pathogen, which as stated above is the causative agent of African tick bite fever in humans [339]. CCHFV, DHOV, NRIV, and THOV can be transmitted from ticks to humans and present as hemorrhagic fever, and occasionally meningoencephalitis [340–342]. Like other *Anaplasma* spp., *A. bovis* and *A. platys* cause disease characterized by anemia and weight loss within affected animals [343]. *Babesia microti*, the primary cause of human babesiosis, produced a malaria-like illness with more severe manifestations in immunocompromised patients such as ARDS, anemia, and disseminated intravascular coagulopathy (DIC) [344]. BHAV is associated with febrile illness and central nervous system manifestations [345]. Rickettsial pox, caused by infection with *R. akari,* is characterized by flu-like symptoms and vesicular lesions on the trunk and extremities [346]. The NSD virus has a mortality rate of up to 90% in non-immune animals and is characterized by hemorrhagic gastroenteritis and abortion [347]. *Ehrlichia canis* causes canine ehrlichiosis, which can progress to severe disease with symptoms of hemorrhage, epistaxis, and shock [348]. *Ehrlichia chaffeensis* is the causative agent of human monocytic ehrlichiosis (HME), with symptoms ranging from vomiting and diarrhea to multiple organ failure [349]. Finally, *B. burgdorferi*, the pathogen responsible for Lyme borreliosis (Lescot et al., 2008), was also detected in Kenya [350]. Multiple pathogens reported within this systematic review are of clinical relevance to both humans and animals [350, 351], impacting public health, food security, and local economies. Data generated by this study provide a critical baseline of tick and TBD surveillance data published from Kenya. However, our study reveals critical gaps in surveillance coverage in the eastern regions of Kenya, which should be targeted for future surveillance efforts.

### Tanzania

A total of 729 unique tick collection events were reported from Tanzania, with only 375 entries including specific geographical information concerning the collection event. Ticks were collected primarily from animals, with over 100 different host species sampled. In total, nine genera of ticks were recorded: *Amblyomma*, *Argas*, *Dermacentor*, *Haemaphysalis*, *Hyalomma*, *Ixodes*, *Nuttalliella*, *Ornithodoros*, and *Rhipicephalus*.

Only 10 articles conducted screening for microbial species present within ticks. Agents found included *A. marginale*, *A. bovis*, *Borrelia duttonii*, *T. equi*, and *T. parva*. *Anaplasma marginale* was the most frequently detected pathogen, which as mentioned above can cause bovine anaplasmosis. Tick-borne relapsing fever is associated with *Borrelia* spp. that are vectored by soft ticks from the genus *Ornithodoros*, and there is evidence that this is a circulating zoonosis within East Africa [352]. Much of Tanzania’s land use is dedicated to agriculture, with livestock raised predominantly by small-scale independent farmers in rural areas [353]. Given the large number of human–animal interactions and the impact TBD can have on livestock animals, there is concern regarding the possible threat ticks pose for the livestock owners, which demonstrates the need for more tick and TBD surveillance to ensure food and economic security. Of note, there were no detailed records which examined tick species or TBD specifically from Zanzibar. Given this is a high-traffic trade island off the coast of mainland Tanzania, it would be worthwhile to survey this area in the future and compare the tick diversity between the two regions. There were few collection records which referenced the southernmost regions of Tanzania—Lindi, Mtwara, and Ruvuma. Further surveillance is needed to address gaps in data between different regions of Tanzania, while further testing of ticks needs to be conducted to understand TBD prevalence.

### Uganda

Our results indicate that there are several opportunities to expand our knowledge of ticks in Uganda. Most tick collection events reported from Uganda were collected from a host animal, with only 7.6% of collection event entries documenting environmental samples collected on vegetation or in burrows or caves. Only 10 of the articles reviewed conducted pathogen testing on collected ticks. Yet in these few studies, *A. marginale*, *E. ruminantium*, *R. africae*, *R. conorii*, and *T. parva* have all been detected, with *E. ruminantium*, the causative agent of heartwater, and *R. africae*, the causative agent of tick bite fever, being the most frequently detected agents. Additional tick surveillance implementing environmental sampling would yield more data characterizing suitable questing habitat within Uganda for each species. In addition, sampling a wider variety of host animals to include more reptiles and birds may yield additional tick taxa not detected in this review. Finally, any future surveillance efforts in Uganda should include pathogen screening and molecular confirmation of tick species identification.

## Limitations

By using the search criteria established for this literature review, there may have been articles that were published in languages other than English and French, and thus were not captured in the initial search results. This is of particular importance for Tanzania, given that a few published studies were noted to have been written in German. For example, a published tick record in Tanzania demonstrated the first recording of *Babesia trautmanni* in 1914, but the language in which the article was published resulted in it not being included. Another limitation of this review was the lack of standardized tick identification methods available. Most studies relied primarily on morphology for tick identification, which may have led to misidentified species. Additionally, since the publication of many of these articles, there have been tick species whose taxonomic classifications have changed over time, which may complicate the accuracy of final species designation within this study. Given frequent movement of animals within the study region, animals surveyed for tick studies may represent animals from other regions, possibly impacting the accuracy of the recorded geographical location of reported tick species and any associated microbial agents. This complicates the assessment of whether the ticks or TBD reported from a given study are representative of the study country or neighboring countries. Additionally, throughout our study we did not differentiate the reported recordings of TBDs according to their endemic or epidemic status.

## Conclusions

This systematic review provides a novel dataset on ticks, their associated microbial organisms, and their geographical occurrences within six countries of Africa. This database is made freely available to the public, and its use is encouraged for those working to mitigate the risk of TBD in Africa. Additionally, records generated by this project contain verbatim information that can be independently scrutinized by users to determine their relevance to future studies. The aggregation of such data allows for trends in the distribution of ticks and TBD over time to be correlated with changes in land use, population growth, and the effects resulting from climate change. This study also highlights the substantial gaps in knowledge regarding the distribution of ticks and TBDs in Central and East Africa. While around 120 articles were sourced for Ethiopia and Kenya, considerably fewer were available for Chad, Djibouti, Tanzania, and Uganda, clearly demonstrating the presence of major surveillance gaps within these countries. Improvement of surveillance coverage within these countries requires sustained investment and is contingent on local scientists being adequately trained to conduct rigorous surveillance that produces high-quality tick collection data. Ensuring that local scientists have access to standardized guidelines and protocols to aid in their sampling strategies is equally important. Sampling can differ depending on the environment and situation, with some sampling conducted for more routine surveillance while other sampling can be targeted in response to ongoing outbreaks. Special emphasis also needs to be placed on confirming details of the geographical origin of surveyed animals, when possible, to avoid any uncertainties that may arise from data collected from animals that have been moved across country borders. Databases such as VectorMap can be used as a template for local scientists to inform them and their studies of what data should be recorded with each tick collection event, to continue building this database into the future. Additionally, testing of ticks for microbial agents needs to be encouraged to improve the current knowledge on the presence and distribution of TBD within the region. As research continues, there is a need for capacity-building at the local level, to ensure that the work is carried out in a systematic and effective manner that continues to build on results of the past. Many of the knowledge gaps identified within this systematic review were in relation to quality and quantity of tick surveillance data. This review highlights the need for additional capacity-building within the studied countries in order to promote acquisition of high-quality data which can be used within databases such as VectorMap to obtain a better understanding of the density and diversity of tick populations. This high-quality data will also allow groups to further highlight the challenges certain communities face regarding the impact of TBDs on the health and well-being of humans, livestock, and wildlife.

### Supplementary Information

**Additional file 1: Figure S1.** PRISMA checklist.

**Additional file 2: Dataset S1.** Excel sheet of all articles and data.

**Additional file 3: Table S1.** References listed by country

## Data Availability

Tick distribution data points for associated pathogens and details of associated collections are openly shared and downloadable from the VectorMap site (vectormap.si.edu).

## Abbreviations

TBD: Tick-borne disease
GDP: Gross domestic product
CCHF: Crimean Congo hemorrhagic fever
GIS: Geographic information system
DUGV: Dugbe virus
JOSV: Jos virus
THOV: Thogoto virus
DHOV: Dhori virus
KADV: Kadam virus
KUPV: Kupe virus
NSD: Nairobi sheep disease
NRIV: Ngari virus
EP: Equine piroplasmosis
HGA: Human granulocytic anaplasmosis
ARDS: Acute respiratory distress syndrome
DIC: Disseminated intravascular coagulopathy
HME: Human monocytic ehrlichiosis
AHF: Alkhurma virus
BHAV: Bhanja virus
KSIV: Karai virus
FMDV: Foot-and-mouth disease virus
MCOV: Marco virus
TIMV: Timbo virus
CHOV: Chaco virus
TBRF: Tick-borne relapsing fever
ATBF: African tick bite fever
BF: Boutonneuse fever
ECF: East coast fever
