# Supplementary material for: Data release: targeted systematic literature search for tick and tick-borne pathogen distributions in six countries in sub-Saharan Africa from 1901 to 2020
Source: Parasit Vectors. 2024 Feb 22;17:84. doi: 10.1186/s13071-023-06086-4 (PMC10885379; doi:10.1186/s13071-023-06086-4)
Supplement: Supplementary file 3 — Additional file 3: Table S1. References listed by country [file 13071_2023_6086_MOESM3_ESM.docx]

**Table 3.** Publications containing georeferenced tick species by country

| Citation | Country/Countries |
| --- | --- |
| Dahmana, H., Amanzougaghene, N., Davoust, B., Normand, T., Carette, O., Demoncheaux, J. P., ... & Mediannikov, O. (2019). Great diversity of Piroplasmida in Equidae in Africa and Europe, including potential new species. *Veterinary Parasitology: Regional Studies and Reports*, 18, 100332. | Chad |
| Mura, A., Socolovschi, C., Ginesta, J., Lafrance, B., Magnan, S., Rolain, J.M., Davoust, B., Raoult, D., Parola, P. (2008). Molecular detection of spotted fever group rickettsiae in ticks from Ethiopia and Chad. *Transactions of the Royal Society of Tropical Medicine and Hygiene*, 102,945–949. | Chad, Djibouti |
| Rodrigues, R., Telles, J. N., Essere, K., Ducournau, C., Roqueplo, C., Levieuge, A., Davoust, B., Parola, P., Paranhos-Baccalà, G., & Peyrefitte, C. N. (2011). Development of a one step real time RT-PCR assay to detect and quantify Dugbe virus. *Journal of Virological* *Methods*, 176(1–2), 74–77. | Chad |
| Trape, J. F., Diatta, G., Arnathau, C., Bitam, I., Sarih, M. H., Belghyti, D., ... & Renaud, F. (2013). The epidemiology and geographic distribution of relapsing fever borreliosis in West and North Africa, with a review of the *Ornithodoros* *erraticus* complex (Acari: Ixodida). *PLoS One*, 8(11), e78473. | Chad |
| Zachée, B., Mahamat, O., Saboune, M., & Julius, A. N. (2020). Prevalence, intensity and risk factors of tick infestation of cattle in N'djamena Chad. *International Journal of Veterinary Sciences and Animal Husbandry*, 5(4), 139–146. | Chad |
| Hoogstraal, H. (1953). On ticks (Ixodidae) of southern French Somaliland and the rediscovery of *Rhipicephalus longicoxatus* Neumann 1905. *Annals Entomological Society of America*, 46, 393–398 | Djibouti |
| Horton, K. C., Fahmy, N. T., Watany, N., Zayed, A., Mohamed, A., Ahmed, A. A., Rollin, P. E., & Dueger, E. L. (2016). Crimean Congo Hemorrhagic Fever virus and Alkhurma (Alkhumra) virus in Ticks in Djibouti. *Vector Borne and Zoonotic Diseases* (Larchmont, N.Y.), 16(10), 680–682. | Djibouti |
| Horton, K. C., Jiang, J., Maina, A., Dueger, E., Zayed, A., Ahmed, A. A., Pimentel, G., & Richards, A. L. (2016). Evidence of Rickettsia and Orientia Infections Among Abattoir Workers in Djibouti. *The American Journal of Tropical Medicine and Hygiene*, 95(2), 462–465. | Djibouti |
| Morel, P. C., & Vassiliades, G. (1962). Les *Rhipicephalus* du groupe *sanguineus*: espèces *africaines* (Acariens: Ixodoidea). *Revue d’élevage et de Médecine Vétérinaire des Pays Tropicaux*, 15(4), 343–386. | Djibouti, Uganda, Kenya, Chad, Ethiopia |
| Morel, P.C. Mouchet, J. Rodhain, F. (1976). Description de *Rhipicephalus* *camicasi* n. sp. (Acariens, Ixodida) des steppes subdésertiques. de la plaine afar. *Revue d’élevage et de Médecine Vétérinaire des Pays Tropicaux*, 29(4), 337–340. | Djibouti |
| Mouchet J. (1971). *Aedes aegypti* and potential vectors of yellow fever in the Democratic Republic of Somalia and in the French Territory of Afars and Issas. *Bulletin of the World Health Organization*. 45(3), 383–394. | Djibouti |
| Rodhain F. (1976). Preliminary results of an entomological survey of the potential arbovirus vectors in the French Territory of Afars and Issas. *Bulletin de la Societe de Pathologie Exotique*. 69(2), 169–174. | Djibouti |
| Socolovschi C, Matsumoto K, Marie JL, Davoust B, Raoult D, Parola P. (2007). Identification of Rickettsiae, Uganda and Djibouti. *Emerging Infectious Diseases*. 13(10), 1508–1510. | Djibouti, Uganda |
| Abdela, N., Ibrahim, N., & Begna, F. (2018). Prevalence, risk factors and vectors identification of bovine anaplasmosis and babesiosis in and around Jimma town, Southwestern Ethiopia. *Acta Tropica*, 177(1),9–18. | Ethiopia |
| Abdella, A., Yimer, M., & Hiko, A. (2017). Prevalence and risk factors of ticks infesting cattle reared on the main campus of Haramaya University, Eastern Ethiopia. *Ethiopian Veterinary Journal*, 21(1), 16–28. | Ethiopia |
| Abebe, R., Fantahun, T., Abera, M., & Bekele, J. (2010) Survey of ticks (Acari: Ixodidae) infesting cattle in two districts of Somali Regional State, Ethiopia. *Veterinary World*, 3(12), 539–543. | Ethiopia |
| Abebe, R., Tatek, M., Megersa, B., & Sheferaw, D. (2011). Prevalence of small ruminant ectoparasites and associated risk factors in selected districts of Tigray Region, Ethiopia. *Global Veterinaria*, 7(5), 433–437. | Ethiopia |
| Abera, A., & Gebrewahd, T.T. (2019). Prevalence and Risk Factors of Ectoparasites in Small Ruminants in and around Haramaya University, Eastern Oromia Region, Ethiopia. *Ethiopia Veterinary Journal*, 23(1)78–89. | Ethiopia |
| Abera, M., Mohammed, T., Abebe, R., Aragaw, K., & Bekele, J. (2010). Survey of ixodid ticks in domestic ruminants in Bedelle district, Southwestern Ethiopia. *Tropical Animal Health and Production*, 42, 1677–1683. | Ethiopia |
| Aboma, H., Kebede, A., & Abdurahaman, M. (2017). Further Studies on Bovine Ixodide Ticks in and around Bedelle, Southwest Ethiopia. *African Journal of Agricultural Research*, 12(22), 1922–1929 | Ethiopia |
| Abunna, F., Kasasa, D., Shelima, B., Megersa, B., Regassa, A., & Amenu, K. (2009). Survey of tick infestation in small ruminants of Miesso district, West Harergie, Oromia Region, Ethiopia. *Tropical Animal Health and Production*, 41, 969–972. | Ethiopia |
| Abunna, F., Tura, J., & Regassa, A. (2012). Status of Tick Infestation in Small Ruminants of Bedelle District, Oromia Region, Ethiopia. *Global Veterinaria*, 8(5), 459–462. | Ethiopia |
| Akinyi, M. Y., Tung, J., Jeneby, M., Patel, N. B., Altmann, J., & Alberts, S. C. (2013). Role of grooming in reducing tick load in wild baboons (Papio cynocephalus). *Animal Behaviour*, 85(3), 559–568. | Kenya |
| Ali, M., & de Castro, J. J. (1993). Host resistance to ticks (Acari: Ixodidae) in different breeds of cattle at Bako, Ethiopia. *Tropical Animal Health and Production*, 25(4), 215–222. | Ethiopia |
| Amante, M., Hailu, Y., Terefe, G., & Asres, K. (2019). In-vitro louscidal and acaricidal activities of alkaloid of Calpurnia aurea extracts against *Linognathus ovillus* and *Amblyomma variegatum*. *International Journal of Applied and Natural Sciences*, 10(1), 431–437. | Ethiopia |
| Amare, S., Asfaw, Y., & Tolossa, Y. H. (2013). Ectoparasites of sheep and goats in north-west Amhara regional state, Ethiopia. *Ethiopian Veterinary Journal*, 17(1), 55–67. | Ethiopia |
| Amoo, A.O.J., Dipeolu, O. O., Capstick, P. B., Munyinyi, D. M., Gichuru, L. N., & Odhiambo, T.R. (1993). Ixodid Ticks (Acari: Ixodidae) and Livestock Production: Effect of Varying Acaricide Treatments on Ticks and Productivity in East Coast Fever-Immunized Weaner and Dairy Cattle. *Journal of Medical Entomology*. 30(3), 503–512. | Kenya |
| Apanaskevich, D. A., & Tomlinson, J. A. (2019). Description of four new species of *Haemaphysalis* Koch, 1844 (Acari: Ixodidae) from the *H*.(*Rhipistoma*) spinulosa subgroup, parasites of carnivores and rodents in Africa. *Systematic Parasitology*, 96, 625–657. | Kenya, Tanzania |
| Aragaw, K., Abdella, A., Fekadu, A., Kassaye, A., Hindebu, B., & Sheferaw, D. (2016). Skin associated problems in working donkeys in three districts of Sidama Zone, Southern Ethiopia. *Ethiopian Veterinary Journal*, 20(2), 99–118 | Ethiopia |
| Ashenafi, H., & Yimer, E. (2005). Ectoparasites of local scavenging chickens of central Ethiopia. *SINET: Ethiopian Journal of Science*, 28(1), 69–74. | Ethiopia |
| Asrate, S., & Yalew, A. (2012). Prevalence of cattle tick infestation in and around Haramaya district, Eastern Ethiopia. *Journal or Veterinary Medicine and Animal Health*, 4(6), 84–88. | Ethiopia |
| Ayalew, T., Hailu, Y., & Kumsa, B. (2014). Ixodid ticks infesting cattle in three agroecological zones in central Oromia: species composition, seasonal variation, and control practices. *Comparative Clinical Pathology*, 23, 1103–1110. | Ethiopia |
| Ayana, D., Eshetu, E., Waketole, H., & Abunna, F. (2013). In-vitro acaricidal efficacy evaluation trial of Ixodid ticks at Borana, Ethiopia. *Ethiopian Veterinary Journal*, 17(2), 85–99. | Ethiopia |
| Balinandi, S., Chitimia-Dobler, L., Grandi, G., Nakayiki, T., Kabasa, W., Bbira, J., ... & Mugisha, L. (2020). Morphological and molecular identification of ixodid tick species (Acari: Ixodidae) infesting cattle in Uganda. *Parasitology Research*, 119, 2411–2420. | Uganda |
| Bayisa, D., Berhanu, A., Fentahun, T., & Chanie, M. (2012). Occurrence of Bovine Dermatophilosis in Ambo town, West Shoa Administrative Zone, Ethiopia. *American-Eurasian Journal of Scientific Research*, 7(4), 172–175. | Ethiopia |
| Bayisa, T., Ibrahim, N., & Dargie, M. (2013). Prevalence of Ovine Ectoparasites in and Around Ambo Town, Ethiopia. *Middle-East Journal of Scientific Research*, 16(1), 62–67. | Ethiopia |
| Bedada, H., Terefe, G., & Tolossa, Y.H. (2015). Current Status of Ectoparasites in Sheep and Management Practices against the Problem in Ectoparasites Controlled and Uncontrolled Areas of Arsi Zone in Oromia Region, Ethiopia. *Journal of Veterinary Science & Technology*, S:10, 002 | Ethiopia |
| Bedasso, M., Abebe, B., & Degefu, H. (2014). Species composition, prevalence and seasonal variations of ixodid cattle ticks in and around Haramaya town, Ethiopia. *Journal of Veterinary Medicine and Animal Health*, 6(5), 131–137. | Ethiopia |
| Bekele, J., Tariku, M., & Abebe, R. (2011). External Parasite Infestations in Small Ruminants in Wolmera District of Oromiya Region, Central Ethiopia. *Journal of Animal and Veterinary Advances*, 10(4), 518–523. | Ethiopia |
| Bekele, T. (2002). Studies on seasonal dynamics of ticks of Ogaden cattle and individual variation in resistance to ticks in eastern Ethiopia. *Journal of Veterinary Medicine*, Series B, 49(6), 285–288. | Ethiopia |
| Belihu, K., Mamo, A., Lobago, F., & Ayana, D. (2009). Prevalence of ectoparasites in backyard local chickens in three agroecologic zones of East Shoa, Ethiopia. *Revue de Médecine Vétérinaire*, 160(11), 537–541. | Ethiopia |
| Beyecha, K., Kumsa, B., & Beyene, D. (2014). Ectoparasites of goats in three agroecologies in central Oromia, Ethiopia. *Comparative Clinical Pathology*, 23, 21–28. | Ethiopia |
| Burgdorfer, W., Schmidt, M. L., & Hoogstraal, H. (1973). Detection of *Trypanosoma theileri* in Ethiopian cattle ticks. *Acta Tropica*, 30 (4), 340-346. | Ethiopia |
| Burgdorfer, W., Ormsbee, R. A., Schmidt, M. L., & Hoogstraal, H. (1973). A search for the epidemic typhus agent in Ethiopian ticks. *Bulletin of the World Health Organization*, 48(5), 563. | Ethiopia |
| Choudhury, M. K., Shiferaw, Y., & Hussen, A. (2015). Toxicity of *Millettia ferruginea darasana* (family: Fabaceae) against the larvae and adult ticks of *Amblyomma variegatum* Fabricius a three-host tick in cattle. *Journal of Parasitic Diseases*, 39, 298–302. | Ethiopia |
| Culter, S., Abdissa, A., Adamu, H., Tolosa, T., & Gashaw, A. (2012). *Borrelia* in Ethiopian ticks. *Ticks and Tick-borne Diseases*, 3, 14–17. | Ethiopia |
| Golo, D., Wubishet, Z., Tadelle, S., Kula, J., Gete, G., & Garu, L. (2017). Composition, prevalence and abundance of Ixodid cattle ticks at Ethio-Kenyan Border, Dillo district of Borana Zone, southern Ethiopia. *Journal of Veterinary Medicine and Animal Health*, 9(8), 204–212. | Ethiopia |
| Dinka, A., Bedada, B, Yacob, H.T. (2010). Study on Major Parasitic Problems of Rural Cattle in and Around Ambo, Western Oromia, Ethiopia. *Nigerian Veterinary Journal*, 31(3), 208–213. | Ethiopia |
| Dinka, A., Eyerusalem, B., & Yacob, H. T. (2010). A study on major ectoparasites of camel in and around Dire Dawa, Eastern Ethiopia. *Revue de Médecine Véterinaire*, 161(11), 498–501. | Ethiopia |
| Eddie B., Foster, W.A., Rodovsky, F.J., Stiller, D. (1970). Isolation of a PL Agent (Chlamydia, Bedsonia) from ticks (*Agras* (*P*.) *arboreus*) parasitic on the white-necked cormorant (phalacrocorax carbo) in Ethiopia. *Journal of Medical Entomology*, 7(6), 745–746. | Ethiopia |
| Eyob, E., & Matios, L. (2014). Preliminary Survey on the Distribution of Ixodid Ticks in Small ruminants of Dhas District or Borena pastoral area, Southern Rangelands of Ethiopia. *Advances in Bioresearch*, 5(1), 87–91. | Ethiopia |
| Fantahun, B., & Mohamed, A. (2012). Survey on the Distribution of Tick Species in and Around Assosa Town, Ethiopia. *Research Journal of Veterinary Medicine*, 5(2), 34–41. | Ethiopia |
| Feleke, A., Petros, B., Lemecha, H., Wossene, A., Mulatu, W., & Rege, E. J. (2008). Study on monthly dynamics of ticks and seroprevalence of *Anaplasma* *marginale*, *Babesia bigemina* and *Theileria mutans* in four indigenous breeds of cattle in Ghibe Valley, Ethiopia. *SINET: Ethiopian Journal of Science*, 31(1), 11–20. | Ethiopia |
| Feleke, A., Petros, B., Mulatu, W., Lemecha, H., Wossene, A., & Rege, J. O. (2007). Resistance of Abigar, Guraghe, Horro and Sheko Breeds of Cattle to Tick Infestation in Ghibe–Tolley Valley. *Bulletin of Animal Health and Production in Africa*, 55(3), 163–174. | Ethiopia |
| Fentahun, T., Woldemariam, F., Chanie, M., and Berhan, M. (2012). Prevalence of Ectoparasites on Small Ruminants in and around Gondar Town. *American-Eurasian Journal of Scientific Research*, 7(3), 106–111. | Ethiopia |
| Ferede, B., Kumsa, B., Bsrat, A., & Kalayou, S. (2010). Ticks of donkeys in central Oromia regional state, Ethiopia. *Revue de Médecine Vétérinaire*, 161(3), 121–126. | Ethiopia |
| Ferede, Y., Mola, L., & Asmare, Z. (2018). Prevalence and species composition of major internal and external parasites of calves in selected dairy farms of Bahir Dar milk-shade. Ethiopian Veterinary Journal, 22(2), 128–142. | Ethiopia |
| Gashaw, A. (2005). Host Preference and Seasonal Variation of Tick (Amblyomma cohaerens Donitz, 1909) on Naturally Infested Cattle in Jimma Zone, Southwestern Ethiopia. *Journal of Agriculture and Rural Development in the Tropics and Subtropics*, 106(1), 49–57. | Ethiopia |
| Gedilu, M., Mohamed, A., & Kechero, Y. (2014). Determination of the Prevelence of Ixodid Ticks of Cattle Breeds, Their Predilection Sites of Variation and Tick Burden Between Different Risk Factors in Bahir Dar, Ethiopia. *Global Veterinaria*, 13(4), 520–529. | Ethiopia |
| Hadgu, M., Taddele, H., Girma, A., Abrha, H., & Hagos, H. (2018). Prevalence of ixodid ticks infesting Raya cattle breeds in Semi-arid areas of Raya Azebo district, northern Ethiopia. *Ethiopian Veterinary Journal*, 22(2), 53–64. | Ethiopia |
| Hiluf, G., Bsrat, A., Kebede, E., & Hagos, Y. (2018). Prevalence and identification of ectoparasites on indigenous chickens in Seharti-Samre District, Tigray, Northern Ethiopia. *Ethiopian Veterinary Journal*, 22(1), 1–10. | Ethiopia |
| Hilina, B., Berihun, A., & Yasmin, J. (2012). Prevalence and identification of ticks in cattle in and around Mekelle. *Revista Electronica de Veterinaria*, 13(9). | Ethiopia |
| Hornok, S., Abichu, G., Meli, M.L., Tanczos, B., Sulyok, K.M., Gyuranecz, M., Gonczi, E., Farkas, R., Hofmann-Lehmann, R. 2014. Influence of the Biotope on the Tick Infestation of Cattle and on the Tick-Borne Pathogen Repertoir of Cattle Ticks in Ethiopia. *PLoS One*, 9(9), e106452. | Ethiopia |
| Hornok, S., Abichu, G., Takacs, N., Gyuranecz, M., Farkas, R., Fernandez de Mera, I., de la Fuente, J. (2016). Molecular Screening for Anaplasmataceae in Ticks and Tsetse flies from Ethiopia. *Acta Veterinaria Hungarica*, 64(1), 65–70. | Ethiopia |
| Isreal, Y., Abera, T., Wakayo, B.U. (2015). Epidemiologicial study on ectoparasite infestation of small ruminants in Sodo Zuria District, Southern Ethiopia. *Journal of Veterinary Medicine and Animal Health*, 7(4), 140–144. | Ethiopia |
| Kassa, S.A. & Yalew, A. 2012. Identification of Ixodide ticks of cattle in and around Hararamaya district, Eastern Ethiopia. *Scientific Journal of Crop Science*, 1(1), 32–38. | Ethiopia |
| Kassaye, E., Moser, I., & Woldemeskel, M. (2003). Epidemiological study on clinical bovine dermatophilosis in northern Ethiopia. DTW. *Deutsche tierarztliche Wochenschrift*, 110(10), 422–425. | Ethiopia |
| Kebede, N., & Fetene, T. (2012). Population dynamics of cattle ectoparasites in Western Amhara National Regional State, Ethiopia. *Journal of Veterinary Medicine and Animal Health*, 4(1), 22–26. | Ethiopia |
| Kemal, J., & Abera, T. (2017). Prevalence and infestation load of ixodid ticks of cattle in Dassenech district, southern Ethiopia. *Ethiopian Veterinary Journal*, 21(2), 121–130. | Ethiopia |
| Kemal, J., Muktar, Y., Alemu, S. Distribution and prevalence of tick infestation in cattle in Babille district, eastern Ethiopia. *Livestock Research for Rural Development*, 28(12), 232 | Ethiopia |
| Kemal, J., Tamerat, N., & Tuluka, T. (2016). Infestation and identification of ixodid tick in cattle: The case of Arbegona District, southern Ethiopia. *Journal of Veterinary Medicine, 1,* 9618291 | Ethiopia |
| Kigaye, M.K., Jiffar, T. (1991). A Survey of Ectoparasites of Cattle in Harar and Dire Dawa Districts, Hararghe Administrative Region of Ethiopia. *Bulletin of Animal Health and Production in Africa*, 39,15–24. | Ethiopia |
| Kumsa, B., Abiy, Y., Abunna, F. (2019). Ectoparasites infesting dogs and cats in Bishoftu, central Oromia, Ethiopia. *Veterinary Parasitology: Regional Studies and Reports*, 15, 100263. | Ethiopia |
| Kumsa, B., Beyecha, K., Geloye, M. Ectoparasites of sheep in three agro-ecological zones in central Oromia, Ethiopia. *Onderstepoort Journal of Veterinary Research*, 79(1), 1–7. | Ethiopia |
| Kumsa, B. E., & Mekonnen, S. (2011). Ixodid ticks, fleas and lice infesting dogs and cats in Hawassa, southern Ethiopia. *Onderstepoort Journal of Veterinary Researc*h, 78(1), 1–4. | Ethiopia |
| Kumsa, B., Signorini, M., Teshale, S., Tessarin, C., Duguma, R., Ayana, D., ... & Cassini, R. (2014). Molecular detection of piroplasms in ixodid ticks infesting cattle and sheep in western Oromia, Ethiopia. *Tropical Animal Health and Production*, 46, 27–31. | Ethiopia |
| Kumsa, B., Socolovschi, C., Roault, D., Parola, P. (2015). Spotted fever group rickettsiae in ixodid ticks in Oromia, Ethiopia. *Ticks & Tick-borne Diseases*, 6, 8–15. | Ethiopia |
| Kumsa, B., Tamrat, H., Tadesse, G., Aklilu, N., Cassini, R. (2012). Prevalence and species composition of ixodid ticks infesting horses in three agroecologies in central Oromia, Ethiopia. *Tropical Animal Health and Production*, 44, 119–124. | Ethiopia |
| Leul, B., Berihun, A., Etsay, K. (2020). Epidemiological Distribution of Major Ectoparasites Species of Small Ruminant in the Case of Chemical Control Compaign in Welkait District, Tigray Region, Ethiopia. *Journal of Tropical Medicine*, 1, 4175842. | Ethiopia |
| Mediannikov, O., Abdissa, A., Socolovschi, C., Diatta, G., Trape, J.F., & Raoult, D. (2013). Detection of a new *Borrelia* species in ticks taken from cattle in Southwest Ethiopia. *Vector-Borne and Zoonotic Diseases*, 13(4), 266–269. | Ethiopia |
| Megersa, B., Damena, A., Bekele, J., Adane, B., Sheferaw, D. (2012). Ticks and mange mites infesting camels of Boran pastoral areas and the associated risk factors, southern Ethiopia. *Journal of Veterinary Medicine and Animal Health*, 4(5):71–77. | Ethiopia |
| MeKonnen, S., Hussein, I., & Bedane, B. (2001). The distribution of ixodid ticks (Acari: Ixodidae) in Central Ethiopia. *Onderstepoort Journal of Veterinary Research*, 68, 243–251. | Ethiopia |
| Mekuria, S., & Gexahegan, E. (2010). Prevalence of external parasite of poultry in intensive and backyard chicken farm at Wolayta Soddo town, Southern Ethiopia. *Veterinary World*, 3(12), 533–538. | Ethiopia |
| Moges, N., Bogale, B., Fentahun, T. (2012). Hard Ticks (Ixodidae): Species composition, seasonal dynamics and body site distribution on cattle in Chilga District, Northwest Ethiopia. *Asian Journal of Agricultural Science*, 4(5), 341–345. | Ethiopia |
| Mulugeta, Y., Yacob, H.T., Ashenafi, H. (2010). Ectoparasites of small ruminants in three selected agro-ecological sites of Tigray Region, Ethiopia. *Tropical Animal Health and Production*, 42, 1219–1224. | Ethiopia |
| Olkeba, W. G., Sarba, E. J., Deres, B. A., & Zewdu, E. (2016). Prevalence of major skin diseases of cattle and associated risk factors around Ambo town, Ethiopia. *Animal Health and Production*, 64, 355–365. | Ethiopia |
| Onu, S. H., & Shiferaw, T. Z. (2013). Prevalence of ectoparasite infestations of cattle in Bench Maji zone, southwest Ethiopia. *Veterinary World*, 6(6), 291–294. | Ethiopia |
| Pegram, R. G., Hoogstraal, H., & Wassef, H. Y. (1981). Ticks (Acari: Ixodoidea) of Ethiopia. I. Distribution, ecology and host relationships of species infesting livestock. *Bulletin of Entomological Research*, 71(2), 339–359. | Ethiopia |
| Philip, C. B., Hoogstraal, H., Reiss-Gutfreund, R., & Clifford, C. M. (1966). Evidence of rickettsial disease agents in ticks from Ethiopian cattle. *Bulletin of the World Health Organization*, 35(2), 127–131. | Ethiopia |
| Regasa, T.D., Tsegay, A.K., Waktole, H. (2015). Prevalence of major ectoparasites of calves and associated risk factors in and around Bishoftu town. *African Journal of Agricultural Research*, 10(10), 1127–1135. | Ethiopia |
| Regassa A., Awol, N., Hadush, B., Tsegaye, Y., Sori, T. (2015). Internal and external parasites of camels (*Camelus dromedarius*) slaughtered at Addis Ababa Abattoir, Ethiopia. *Journal of Veterinary Medicine and Animal Health*, 6(7), 57–63. | Ethiopia |
| Regassa, A., & De Castro, J. J. (1993). Tick resistance to acaricides in western Ethiopia. *Tropical Animal Health and Production*, 25(2), 69–74. | Ethiopia |
| Regassa, A. (2001). Tick Infestation of Borana cattle in the Borana Province of Ethiopia. *Onderstepoort Journal of Veterinary Research*, 68, 41–45. | Ethiopia |
| Seid, M., Zeryehun, T., Kemal, J., & Tilahun, B. (2018). Ectoparasites of small ruminants in and around Kombolcha, northeastern Ethiopia. *Ethiopian Veterinary Journal*, 22(2), 81–93. | Ethiopia |
| Sertse, T., Wossene, A. (2007). A study on ectoparasites of sheep and goats in eastern part of Amhara region, northeast Ethiopia. *Small Ruminant Research*, 69,62–67. | Ethiopia |
| Seyoum, Z., Tadesse, T., Addisu, A. (2015). Ectoparasites Prevalence in Small Ruminants in and around Sekela, Amhara Regional State, Northwest Ethiopia. *Journal of Veterinary Medicine*, 1, 216085. | Ethiopia |
| Sheferaw, D. (2017). Tick resistance of two breeds of cattle in Wolaita Zone, Southern Ethiopia. *Journal of Veterinary Medicine and Animal Health*; 9(12),349–355. | Ethiopia |
| Siyoun, T., & Kitaw, G. (2014). Comparative milk production and prevalence study of parasites and sub clinical mastitis on indigenous lactating cows under different feeding regimes in central highlands of Ethiopia. *Ethiopian Veterinary Journal*, 18(1), 43–56. | Ethiopia |
| Solomon, G. & Kaaya, G.P. (1996). Comparison of resistance in three breeds of cattle against African ixodid ticks. *Experimental & Applied Acarology*, 20, 223–230. | Ethiopia |
| Solomon, G., Kaaya, G. P., Gebreab, F., Gemetchu, T., & Tilahun, G. (1998). Ticks and tick-borne parasites associated with indigenous cattle in Didtuyura ranch, Southern Ethiopia. *International Journal of Tropical Insect Science*, 18(1), 59–66. | Ethiopia |
| Sulyok, K.M., Hornok, S., Abichu, G., Erdelyi, K., Gyuranecz. (2014). Identification of novel Coxiella burnettii genotypes from Ethiopian ticks. *PLos One*, 9(11), e113213. | Ethiopia |
| Tadesse, A., Fentaw, E., Mekbib, B., Abebe, R., Mekuria, S., & Zewdu, E. (2011). Study on the prevalence of ectoparasite infestation of ruminants in and around Kombolcha and damage to fresh goat pelts and wet blue (pickled) skin at Kombolch Tannary, Northeastern Ethiopia. *Ethiopian Veterinary Journal*, 15(2), 87–101. | Ethiopia |
| Tadesse, B., & Sultan, A. (2014). Prevalence and distribution of tick infestation on cattle at Fitche Selale, North Shewa, Ethiopia. *Livestock Research for Rural Development*, 28(8),1–8. | Ethiopia |
| Tadesse, F., Abadfaji, G., Girma, S., Kumsa, B., & Jibat, T. (2012). Identification of tick species and their perferred site on cattle's body in and around Mizan Teferi, Southwestern Ethiopia. *Journal of Veterinary Medicine and Animal Health*, 4(1),1–5. | Ethiopia |
| Tafesse, B. (1996). Survey on the distribution of ticks of domestic animals in the eastern zone of Ethiopia. *Tropical Animal Health and Production*, 28, 145–146. | Ethiopia |
| Tamerat N, Korso L, Mengistu S, Muktar Y, Keffale M. (2016). Prevalence and identification of ectoparasites fauna in small ruminants in and around Adami Tulu, East Shawa zone of Oromia, Ethiopia. *Livestock Research for Rural Development*, 28(11). | Ethiopia |
| Tesfaheywet, Z., & Simeon, H. (2016). Major ectoparasites of small ruminants in Bench Maji Zone, southern Ethiopia. *Livestock Research for Rural Development*, 28(4), 63. | Ethiopia |
| Tesfaye, A. & Chanie, M. (2011). Ectoparasites are Major Skin Diseases of Dogs in Gondar, Amhara National Regional State, Ethiopia, *International Journal of Animal and Veterinary Advances*, 3(5), 392–396. | Ethiopia |
| Tesfaye, D., Assefa, M., Demissie, T. & Taye, M. (2012). Ectoparasites of small ruminants presented at Bahir Dar Veterinary Clinic, Northwest Ethiopia. *African Journal of Agricultural Research*, 7(33), 4669–4674. | Ethiopia |
| Teshale, S., Geysen, D., Ameni G., Ketema, B., Dorny, P., & Berkvens, D. (2016). Molecular Detection of *Anaplasma* species in questing ticks (ixodids) in Ethiopia. *Asian Pacific Journal of Tropical Disease*, 6(6), 449–452. | Ethiopia |
| Teshale, S., Geysen, D., Ameni, G., Asfaw, Y., Berkvens, D. (2015). Improved molecular detection of Ehrlichia and *Anaplasma* species applied to *Amblyomma* ticks collected from cattle and sheep in Ethiopia. *Ticks & Tick-borne Diseases*, 6, 1–7. | Ethiopia |
| Teshale, S., Kumsa, B., Menandro, M.L., Cassini, R., Martini, M. (2016). *Anaplasma*, *Ehrlichia* and rickettsial pathogens in ixodid ticks infesting cattle and sheep in western Oromia, Ethiopia. *Experimental and Applied Acarology*, 70, 231–237. | Ethiopia |
| Teshome, D. (2016). Prevalence of major skin diseases in ruminants and its associated risk factors at University of Gondar Veterinary Clinic, North West Ethiopia. *Journal of Research and Development*, 4(1), 1–7. | Ethiopia |
| Tessema, T., & Gashaw, A. (2010). Prevalence of ticks on local and crossbred cattle in and around Asella town, southeast Ethiopia. *Ethiopian Veterinary Journal*, 14(2), 79–89. | Ethiopia |
| Tiki, B. & Mekonnen, A. (2011). Distribution of Ixodid Ticks on Cattle in and Around Holeta Town, Ethiopia. *Global Veterinaria*, 7(6),527–531. | Ethiopia |
| Tilki, T., Eshetu, A., & Waktola, H. (2015). Major ectoparasites of cattle in Ada'a district, East Showa Zone, Ethiopia. *Livestock Research for Rural Development*, 27(10), 198. | Ethiopia |
| Tomassone, L., Grego, E., Callà, G., Rodighiero, P., Pressi, G., Gebre, S., ... & De Meneghi, D. (2012). Ticks and tick-borne pathogens in livestock from nomadic herds in the Somali Region, Ethiopia. *Experimental and Applied Acarology*, 56, 391–401. | Ethiopia |
| Wasihun, P., & Doda, D. (2013). Study on prevalence and identification of ticks in Humbo district, Southern Nations, Nationalitites, and People's Region (SNNPR), Ethiopia. *Journal of Veterinary Medicine and Animal Health*, 5(3), 73–80. | Ethiopia |
| Woldemeskel, M., & Mersha, G. (2010). Study on caprine and ovine dermatophilosis in Wollo, Northeast Ethiopia. *Tropical Animal Health and Production*, 42(1), 41. | Ethiopia |
| Wood, O. L., Lee, V. H., Ash, J. S., & Casals, J. (1978). Crimean-Congo hemorrhagic fever, Thogoto, dugbe, and Jos viruses isolated from ixodid ticks in Ethiopia. *The American Journal of Tropical Medicine and Hygiene*, 27(3), 600–604. | Ethiopia |
| Yacob, H. T., Ataklty, H., & Kumsa, B. (2008). Major ectoparasites of cattle in and around Mekelle, northern Ethiopia. *Entomological Research*, 38(2), 126–130. | Ethiopia |
| Yacob, H. T., Yalew, T. A., & Dinka, A. A. (2008). Part I: ectoparasite prevalences in sheep and in goats in and around Wolaita soddo, Southern Ethiopia. *Revue de Médecine Vétérinaire*, 159(8-9), 450–454. | Ethiopia |
| Yehualashet, T., Gebreab, F., Wakjira, A., & Tsega, T. (1995). Preliminary observation on ticks: Seasonal dynamics and resistance of three indigenous and three cross-bred cattle in Ethiopia. *Bulletin of Animal Health and Production in Africa*, 43(2), 105–114. | Ethiopia |
| Yilma, J. M., Daniel, W. S., & Dorchies, P. (1995). Survey of ticks infesting domestic ruminants in South Wollo region of Ethiopia. *Revue de Médecine Vétérinaire*, 146(3), 213–220. | Ethiopia |
| Yilma, J., Adamu, G., & Zerbini, E. (2001). Biossay of acaricide resistance on three common cattle tick species at Holotta, Central Ethiopia. *Revue de Médecine Vétérinaire*, 152(5), 385–390. | Ethiopia |
| Yonas, M., Welegerima, K., Laudisoit, A., Bauer, H., Gebrehiwot, K., Deckers, S., ... & Leirs, H. (2011). Preliminary investigation on rodent–ectoparasite associations in the highlands of Tigray, Northern Ethiopia: implications for potential zoonoses. *Integrative Zoology*, 6(4), 366–374. | Ethiopia |
| Zeleke, M., & Bekele, T. (2004). Species of ticks on camels and their seasonal population dynamics in Eastern Ethiopia. *Tropical Animal Health and Production*, 36(3), 225–231. | Ethiopia |
| Zeru F, Bedad H, Gebru M, Seid A & Gebregergious A. (2015). Epidemiology of Major Small Ruminant Ectoparasites and Effectiveness of the Control Approaches Employed in Selected Pastoral Districts of Afar, Northeastern Ethiopia. *Journal of Biology, Agriculture and Healthcare*, 5(14), 63–73. | Ethiopia |
| Zeryehun, T. & Atomsa, M. (2012). Ectoparasite infestations of sheep and goat. *Eurasian Journal of Veterinary Sciences*, 28(4), 185–189. | Ethiopia |
| Bwangamoi, O. (1972). A report on generalized equine ringworm (*Trichophyton equinum*) complicated by tick infestation (*Boophilus decoloratus*) and Besnoitiosis. *Bulletin of Epizootic Diseases of Africa*, 20(3), 211–220. | Kenya |
| Campana, M. G., Hawkins, M. T., Henson, L. H., Stewardson, K., Young, H. S., Card, L. R., ... & Fleischer, R. C. (2016). Simultaneous identification of host, ectoparasite and pathogen DNA via in‐solution capture. *Molecular Ecology Resources*, 16(5), 1224–1239. | Kenya |
| Chiera, J. W., Newson, R. M., & Karuhize, G. R. (1989). Adaptation of field strains of *Rhipicephalus appendiculatus* Neumann (Acarina: Ixodidae) to host resistance. *Parasitology*, 99(1), 149–155. | Kenya |
| Clifford, C. M., Kohls, G. M., & Hoogstraal, H. (1968). Ixodes walkerae, n. sp., from a bird in Kenya (Agarina: Ixodidae). *Journal of Medical Entomology*, 5(4), 513–514. | Kenya |
| Clifford, C.M., Flux, J.E., Hoogstraal, H. (1976). Seasonal and regional abundance of ticks (Ixodidae) on hares (Leporidae) in Kenya. *Journal of Medical Entomology*, 13(1), 40–47 | Kenya, Uganda |
| D’Amico, G., Dumitrache, M. O., Široký, P., Albrechtová, K., Sloboda, M., Domşa, C., . . . Mihalca, A. D. (2015). Altitudinal and seasonal differences of tick communities in dogs from pastoralist tribes of Northern Kenya. *Veterinary Parasitology*, 212(3-4), 318–323. | Kenya |
| Daubney, R. and Hudson, J.R. (1934). Nairobi sheep disease: natural and experimental transmission by ticks other than *Rhipicephalus appendiculatus*. *Parasitology*, 26(4), 496–509. | Kenya |
| Davies F. G. (1978). Nairobi sheep disease in Kenya. The isolation of virus from sheep and goats, ticks and possible maintenance hosts. The Journal of Hygiene, 81(2), 259–265. | Kenya |
| Davies F. G. (1982). Karai virus, a probable arbovirus isolated from sheep and from the tick *Rhipicephalus evertsi* in Kenya. *Journal of Comparative Pathology*, 92(1), 9–14. | Kenya |
| De Castro, J. J., Young, A. S., Dransfield, R. D., Cunningham, M. P., & Dolan, T. T. (1985). Effects of tick infestation on Boran (Bos indicus) cattle immunized against theileriosis in an endemic area of Kenya. *Research in Veterinary Science*, 39(3), 279–288. | Kenya |
| de Castro, J.J. (1986). Effects of artificial and natural tick infestations on cattle. *Ticks and Tick-borne Diseases*, 17, 113–115 | Kenya |
| DeTray, D.E., Zaphiro, D., Hay, D. (1961). The incidence of African swine fever in wart hogs in Kenya - a preliminary report. *Journal of the American Veterinary Medical Association*, 138(2), 78–80 | Kenya |
| Dioli, M., Jean-Baptiste, S., & Fox, M. (2001). Ticks (Acari: Ixodidae) of the One-Humped Camel (Camelus dromedarius) in Kenya and Southern Ethiopia: Species, Composition, Attachment Sites, Sex Ratio and Season Incidence. *Revue d'élevage et de Médecine Vétérinaire des Pays Tropicaux*.54(2), 115–122. | Kenya, Ethiopia |
| Dolan, R., Wilson, A. J., Schwartz, H. J., Newson, R. M., & Field, C. R. (1983). Camel production in Kenya and its constraints. II. Tick infestation. *Tropical Animal Health and Production*, 15(3), 179–185. | Kenya |
| El Kammah, K. M., Hoogstraal, H., & Camicas, J. L. (1992). Notes on African *Haemaphysalis* ticks: XI. *H*.(*Rhipistoma*) *paraleachi* (Ixodoidea: Ixodidae) distribution and hosts of adults. *International Journal of Acarology*, 18(3), 205–212. | Kenya, Ethiopia, Uganda |
| Fotheringham, W., Lewis, E.A. (1936). East cost fever: its transmission by ticks in Kenya colony. *Parasitology*, 29(4), 504–523 | Kenya |
| Gitao C. G. (1993). The epidemiology and control of camel dermatophilosis. *Revue d'elevage et de Medecine Veterinaire des Pays Tropicaux*, 46(1-2), 309–311. | Kenya |
| Gitau, G. K., Mcdermott, J. J., Katende, J. M., O'callaghan, C. J., Brown, R. N., & Perry, B. D. (2000). Differences in the epidemiology of theileriosis on smallholder dairy farms in contrasting agro-ecological and grazing strata of highland Kenya. *Epidemiology and Infection*, 124(2), 325–335. | Kenya |
| Gregory, M.V. (1981). Diseases and parasites of the Central African hedgehog Erinaceus albiventris Wagner. *Zoologische Beitrage*, 27, 205–213. | Kenya |
| Grootenhuis, J. G., Morrison, W. I., Karstad, L., Sayer, P. D., Young, A. S., Murray, M., & Haller, R. D. (1980). Fatal theileriosis in eland (*Taurotragus oryx*): pathology of natural and experimental cases. *Research in Veterinary Science*, 29(2), 219–229. | Kenya |
| Guerra, A. S., Eckerlin, R. P., Dowling, A. P., Durden, L. A., Robbins, R. G., Dittmar, K., Helgen, K. M., Agwanda, B., Allan, B. F., Hedlund, T., & Young, H. S. (2016). Host-Parasite Associations in Small Mammal Communities in Semiarid Savanna Ecosystems of East Africa. *Journal of Medical Entomology*, 53(4), 851–860. | Kenya |
| Haig, D. A., Woodall, J. P., & Danskin, D. (1965). Thogoto virus: a hitherto undescribed agent isolated from ticks in Kenya. *Microbiology*, 38(3), 389–394. | Kenya |
| Hassan, S., Dipeolu, O., & Munyinyi, D. (1992). Influence of exposure period and management methods on the effectiveness of chickens as predators of ticks infesting cattle. *Veterinary Parasitology*, 43(3-4), 301–309. | Kenya |
| Hassan, Shawgi & Dipeolu, O & Malonza, M. (1994). Natural attraction of livestock ticks by the leaves of a shrub. *Tropical Animal Health and Production*. 26. 87–91. | Kenya |
| Hassan, Shawgi & Dipeolu, O.O. & Amoo, A.O. & Odhiambo, T.R. (1991). Predation on livestock ticks by chickens. *Veterinary Parasitology*. 38. 199–204. | Kenya |
| Heisch RB, Guggisberg CA. On O*rnithodoros graingeri* N.S.P., a tick from caves in Kenya. *Parasitology*. 1953;42(3-4), 192–198. | Kenya |
| Heisch RB, Guggisberg CA. (1952). A description of *Ornithodoros erraticus* (Lucas) from Kenya. *Annals of Tropical Medicine and Parasitology*, 46(1), 1–6. | Kenya |
| Heisch, R.B. (1954). *Argas brumpti* Neumann in the Kitui district of Kenya. *The East African Medical Journal*, 31(10), 483–484. | Kenya |
| Heisch, R.B. (1954). *Ornithodoros moubata* (Murray) in a porcupine burrow near Kitui. *The East African Medical Journal*, 31(10), 483. | Kenya |
| Heisch, R.B., Grainger, W.E., Harvey, A.E.C., and Lister, G. (1962). Feral aspects of rickettsial infections in Kenya. *Transactions of the Royal Society of Tropical Medicine and Hygiene*, 56, 272–282. | Kenya |
| Heisch, R.B., McPhee, R., Rickman, L.R. (1957). The epidemiology of tick-typhus in Nairobi. East African Medical Journal, 34(9), 459-477 and Heisch, R.B., Harvey, A. E. C. (1957). Rickettsioses in Kenya: serological reactions of wild rodents and inoculated guinea pigs. *East African Medical Journal*, 36(2), 116–118. | Kenya |
| Hoogstraal, H., Clifford, C.M., Keirans, J.E. (1979). The *Ornithodoros* (*Alectorobius*) *capensis* group (Acarina: Ixodoidea: Argasidae) of the Palearctic and Oriental regions. *O*. (*A*.) *coniceps* identity, bird and mammal hosts, virus infections, and distribution in Europe, Africa, and Asia. *The Journal of Parasitology*, 65(3), 395–407. | Kenya |
| Hoogstraal, H., El Kammah, K.M., Camicas, J.L. (1992). Notes on African *Haemaphysalis* ticks: XVI. *H*. (*Rhipistoma*) *subterra* sp. N., a new member of the leachi group (Ixodoidea: Ixodidae). *International Journal of Acarology*, 18(3), 213–220. | Kenya, Ethiopia |
| Hoogstraal, H., Kaiser, M.N., Walker, J.B., Ledger, J.A., Converse, J.D., Rice, R.C.A. (1975). Obervations on the subgenus *Argas* (Ixodoidea: Argasidae: Argas). *A*. (*A*.) *africolumbae*, n. sp., a Pretoria virus-infected parasite of birds in southern and eastern Africa. *Journal of Medical Entomology*, 12(2), 194–201. | Kenya |
| Hoogstraal, H., Wassef, H. Y., Easton, E.R., & Dixon, J.E. (1977). Observations on the subgenus *Argas* (Ixodoidea: Argasidae: *Argas*). 12. *Argas* (*A*.) africolumbae: variation, bird hosts, and distribution in Kenya, Tanzania, and South and South-West Africa. *Journal of Medical Entomology*, 13(4-5), 441–445 | Kenya, Tanzania |
| Horak, I. G., Apanaskevich, D. A., & Kariuki, E. K. (2013). A new species of *Rhipicephalus* (Acari: Ixodidae), a Parasite of Giraffes in Kenya. *Journal of Medical Entomology,* 50(4), 685–690. | Kenya |
| Hornok, S., Szőke, K., Meli, M. L., Sándor, A. D., Görföl, T., Estók, P., Wang, Y., Tu, V. T., Kováts, D., Boldogh, S. A., Corduneanu, A., Sulyok, K. M., Gyuranecz, M., Kontschán, J., Takács, N., Halajian, A., Epis, S., & Hofmann-Lehmann, R. (2019). Molecular detection of vector-borne bacteria in bat ticks (Acari: Ixodidae, Argasidae) from eight countries of the Old and New Worlds. *Parasites & Vectors*, 12(1), 50. | Kenya |
| Humke, R. (1973). *Spray race trails with the acaricide Batestan in Kenya*. The Blue Book, 23, 7–16. | Kenya |
| Irvin, A. D., Brown, C. G., Burridge, M. J., Cunningham, M. P., Musoke, A. J., Pierce, M. A., Purnell, R. E., & Radley, D. E. (1972). A pathogenic theilerial syndrome of cattle in the Narok District of Kenya. I. Transmission studies. *Tropical Animal Health and Production*, 4(4), 220–229. | Kenya |
| Irvin, A.D., Sale, J.B., Purnell, R.E. (1973). *Babesia thomasi* from Rock Hyraces in Kenya. *The Journal of Parasitology*, 59(1), 203–204. | Kenya |
| J. C. Njanja, F. G. Ft. Rinkanya & H. K. Kiara (1991) Ticks of camels, sheep and goats in northwestern Kenya rangelands, *Tropical Pest Management*, 37(2), 166–168. | Kenya |
| Johnson, B. K., Chanas, A. C., Squires, E. J., Shockley, P., Simpson, D. I., Parsons, J., Smith, D. H., & Casals, J. (1980). Arbovirus isolations from ixodid ticks infesting livestock, Kano Plain, Kenya. *Transactions of the Royal Society of Tropical Medicine and Hygiene*, 74(6), 732–737. | Kenya |
| Kagira, J. M., Kanyari, P. N., Maingi, N., Githigia, S. M., Ng'ang'a, C., & Gachohi, J. (2013). Relationship between the prevalence of ectoparasites and associated risk factors in free-range pigs in Kenya. *International Scholarly Research Notices*, 2013, 650890. | Kenya |
| Kanduma, E. G., Mwacharo, J. M., Sunter, J. D., Nzuki, I., Mwaura, S., Kinyanjui, P. W., Kibe, M., Heyne, H., Hanotte, O., Skilton, R. A., & Bishop, R. P. (2012). Micro- and minisatellite-expressed sequence tag (EST) markers discriminate between populations of *Rhipicephalus* *appendiculatus*. *Ticks & Tick-borne Diseases*, 3(3), 128–136. | Kenya |
| Kariuki, D. P., Injairo, R., Boyce, W. L., Wellde, B. T., & Ngethe, S. (1989). Parasite survey of eight wild animals in the Ruma National Park. *Annals of Tropical Medicine and Parasitology*, 83(sup1), 115–118. | Kenya |
| Kariuki, D. P., Young, A. S., Morzaria, S. P., Lesan, A. C., Mining, S. K., Omwoyo, P., Wafula, J. L., & Molyneux, D. H. (1995). *Theileria parva* carrier state in naturally infected and artificially immunized cattle. *Tropical Animal Health and Production*, 27(1), 15–25. | Kenya |
| Kariuki, E. K., Penzhorn, B. L., & Horak, I. G. (2012). Ticks (Acari: Ixodidae) infesting cattle and African buffaloes in the Tsavo conservation area, Kenya. *The Onderstepoort Journal of Veterinary Research*, 79(1), E1–E4. | Kenya |
| Kariuki, E., Kutima, H., Kock, M., Horak, I. G., Jooste, R., & Neves, L. (2019). Ixodid ticks (Acari: Ixodidae) collected from African savanna elephants (*Loxodonta africana*) and African forest elephants (*Loxodonta cyclotis*). *The Onderstepoort Journal of Veterinary Research*, 86(1), e1–e5. | Kenya |
| Keesing, F., Allan, B. F., Young, T. P., & Ostfeld, R. S. (2013). Effects of wildlife and cattle on tick abundance in central Kenya. *Ecological Applications*, 23(6), 1410–1418. | Kenya |
| Keesing, F., Ostfeld, R. S., Okanga, S., Huckett, S., Bayles, B. R., Chaplin-Kramer, R., . . . Allan, B. F. (2018). Consequences of integrating livestock and wildlife in an African savanna. *Nature Sustainability*, 1(10), 566–573. | Kenya |
| Keirans, J.E., Hoogstraal, H., Clifford, C.M. (1977). Ornithodoros (Proknekalia) vansomereni, New Subgenus and New Species (Acarina: Ixodoidea: Argasidae), a Swallow Nest Parasite in Kenya. *Annals of the Entomological Society of America*, 70(2), 221–228. | Kenya |
| Kimita, G., Mutai, B., Nyanjom, S. G., Wamunyokoli, F., & Waitumbi, J. (2016). Phylogenetic Variants of *Rickettsia africae*, and Incidental Identification of "*Candidatus Rickettsia moyalensis*" in Kenya. *PLoS Neglected Tropical Diseases*, 10(7), e0004788. | Kenya |
| King'ori, E., Obanda, V., Chiyo, P. I., Soriguer, R. C., Morrondo, P., & Angelone, S. (2019). Molecular identification of *Ehrlichia*, *Anaplasma*, *Babesia* and *Theileria* in African elephants and their ticks. *PLoS One*, 14(12), e0226083. | Kenya |
| Kipronoh, K., Gathuma, J.M., Kitala, P.M., Kiara, H.K. (2011). Prevalence of Tick-borne Infections in Extensive Cattle Management System in West Pokot District, Kenya. *Bulletin of Animal Health and Production in Africa*. 59(1), 43–52. | Kenya |
| Knobel, D. L., Maina, A. N., Cutler, S. J., Ogola, E., Feikin, D. R., Junghae, M., . . . Njenga, M. K. (2013). *Coxiella burnetii* in Humans, Domestic Ruminants, and Ticks in Rural Western Kenya. *The American Journal of Tropical Medicine and Hygiene,* *88*(3), 513–518. | Kenya |
| Koka, H., Sang, R., Kutima, H. L., & Musila, L. (2017). The detection of spotted fever group *rickettsia* DNA in tick samples from pastoral communities in Kenya. *Journal of Medical Entomology*, 54(3), 774–780. | Kenya |
| Koka, H., Sang, R., Kutima, H. L., & Musila, L. (2018). *Coxiella burnetii* Detected in Tick Samples from Pastoral Communities in Kenya. *BioMed Research International,* *2018*, 1–5. | Kenya |
| Kubasu, S.S., Makokkah, G.L., & Kaaya, G. (1971). Biological differences within *Rhipicephalus appendiculatus* Neumann (Acari: Icodidae populations in Kenya. *Journal of the Egyptian Society of Parasitology*, 37(2), 411–418. | Kenya |
| Larkin, P.J. (1961). Control of the Blue Tick (*Boophilus decoloratus*) on Cattle with Pyrethrum Sprays. *The Veterinary Records*, 73(12), 298–300. | Kenya |
| Latif, A.A., Punyua, D.K., Capstick, P.B., & Newson, R.M. (1991). Tick Infestations on Zebu Cattle in Western Kenya: Host Resistance to *Rhipicephalus appendiculatus* (Acari: Ixodidae). *Journal of Medical Entomology*, 28(1), 127–132. | Kenya |
| Lewis, E.A. (1932). *Rhipicephalus ayrei* n.sp. (a tick) from Kenya colony. *Parasitology*, 25(2), 269–272. | Kenya |
| Lohding, G.M. & Young, A.S. (1984). Epidemiology of theilerioses in the Trans-Mara division, Kenya: Husbandry and disease background and preliminary investigations on theilerioses in calves. *Preventative Veterinary Medicine*, 2(6), 801–831. | Kenya |
| Lutomiah, J., Musila, L., Makio, A., Ochieng, C., Koka, H., Chepkorir, E., . . . Sang, R. (2014). Ticks and Tick-Borne Viruses from Livestock Hosts in Arid and Semiarid Regions of the Eastern and Northeastern Parts of Kenya. *Journal of Medical Entomology*, 51(1), 269–277. | Kenya |
| Lwande, O. W., Lutomiah, J., Obanda, V., Gakuya, F., Mutisya, J., Mulwa, F., . . . Sang, R. (2013). Isolation of Tick and Mosquito-Borne Arboviruses from Ticks Sampled from Livestock and Wild Animal Hosts in Ijara District, Kenya. *Vector-Borne and Zoonotic Diseases*, 13(9), 637–642. | Kenya |
| Maamun, J. M., Suleman, M. A., Akinyi, M., Ozwara, H., Kariuki, T., & Carlsson, H. E. (2011). Prevalence of *Babesia microti* in free-ranging baboons and African green monkeys. *The Journal of Parasitology*, 97(1), 63–67. | Kenya |
| Macaluso, K. R., Alam, U., Azad, A. F., Rutherford, J. S., Rosenberg, R., Korman, A., & Davis, J. (2003). Spotted Fever Group Rickettsiae in Ticks From The Masai Mara Region of Kenya. *The American Journal of Tropical Medicine and Hygiene*, 68(5), 551–553. | Kenya |
| Machado-Ferreira E., Vizzoni, V.F., Balsemao-Pires, E., Moerbeck, L., Gazeta, G.S., Piesman, J., Voloch, C.M., Soares, C.A. (2016). *Coxiella* symbionts are widespread into hard ticks. *Parasitology Research*. 115, 4691–4699. | Kenya |
| Maina, A. N., Jiang, J., Omulo, S. A., Cutler, S. J., Ade, F., Ogola, E., Feikin, D. R., Njenga, M. K., Cleaveland, S., Mpoke, S., Ng'ang'a, Z., Breiman, R. F., Knobel, D. L., & Richards, A. L. (2014). High prevalence of Rickettsia africae variants in *Amblyomma variegatum* ticks from domestic mammals in rural western Kenya: implications for human health. *Vector-borne and Zoonotic Diseases*, 14(10), 693–702. | Kenya |
| Moll, G., Lohding, A., Young, A. S., & Leitch, B. L. (1986). Epidemiology of theileriosis in calves in an endemic area of Kenya. *Veterinary Parasitology*, 19(3-4), 255–273. | Kenya |
| Morzaria, S.P., Irvin, A.D., Taracha, E., Spooner, P.R., Voight, W.P., Fujinaga, T., Katende, J. (1987). Immunization against east coast fever: the use of selected stocks of *Theileria parva* for immunization of cattle exposed to field challenge. *Veterinary Parasitology*, 23, 23–41 | Kenya |
| Muenstermann S, Rinkanya FGR, and Tome NR.Tick control in small ruminants with a Cypermethrin ‘pour‐on’ in Kenya (1988). *International Journal of Pest Management,* 34(4), 399–401. | Kenya |
| Mungube, E. O., Bauni, S. M., Tenhagen, B.-A., Wamae, L. W., Nzioka, S. M., Muhammed, L., & Nginyi, J. M. (2007). Prevalence of parasites of the local scavenging chickens in a selected semi-arid zone of Eastern Kenya. Tropical Animal Health and Production, 40(2), 101–109. | Kenya |
| Mungube, E.O., Nzioka, S.M., & Wamae, L.W. (2014). Effect of deltamethrin on Argas persicus within selected sites in Machakos and Kitui Counties, Semi-arid Eastern Kenya. *Livestock Research for Rural Development*. 26(12), 217. | Kenya |
| Muruthi CW, Lwande OW, Makumi JN, Runo S, Otiende M, et al. (2016). Phenotypic and Genotypic Identification of Ticks Sampled from Wildlife Species in Selected Conservation Sites of Kenya. *Journal of Veterinary Science & Technology*, 7 (1), 293. | Kenya |
| Mutai, B. K., Wainaina, J. M., Magiri, C. G., Nganga, J. K., Ithondeka, P. M., Njagi, O. N., Jiang, J., Richards, A. L., & Waitumbi, J. N. (2013). Zoonotic surveillance for rickettsiae in domestic animals in Kenya. *Vector-borne and Zoonotic Diseases*, 13(6), 360–366. | Kenya |
| Mwamuye M.M., Kariuki E., Omondi D., Kabii J., Odongo D., Masiga D. and Villinger J. (2017) Novel Rickettsia and emergent tick-borne pathogens: A molecular survey of ticks and tick-borne pathogens in Shimba Hills National Reserve, Kenya. *Ticks & Tick-Borne Diseases,* 8, 208–218. | Kenya |
| Mwangi, E. N., Newson, R. M., & Kaaya, G. P. (1993). A Hymenopteran Parasitoid of The Bont Tick *Amblyomma variegatum* Fabricius (acarina, Ixodidae) In Kenya. *Discovery and Innovation*, 5(4), 331–335. | Kenya |
| Mwangi, E. N., Sayer, P. D., Njanja, J. C., & Bell, J. F. (1985). Tick survey on goats and sheep in Kenya. Tropical Animal Health and Production, 17(2), 102–106. | Kenya |
| Ndarathi, C. M., Waghela, S., & Semenye, P. P. (1989). Prevalence of ticks on rangeland domestic ruminants in Kajiado district, Kenya. *Bulletin of Animal Health and Production in Africa*, 37(3), 209–212. | Kenya |
| Ndeereh, D., Muchemi, G., Thaiyah, A., Otiende, M., Angelone-Alasaad, S., & Jowers, M. J. (2017). Molecular survey of *Coxiella burnetii* in wildlife and ticks at wildlife-livestock interfaces in Kenya. *Experimental & Applied Acarology*, 72(3), 277–289. | Kenya |
| Newson, R. M. (1978). The life cycle of *Rhipicephalus appendiculatus* on the Kenyan coast. *Tick Borne Diseases and their Vectors;* *Proceedings of an International Conference*., 46–50. | Kenya |
| Obanda, V., Kagira, J., Chege, S., Ouma, B. O., & Gakuya, F. (2011). Trypanosomosis and other co-infections in translocated black (*Diceros bicornis michaeli*) and white (*Ceratotherium simum simum*) rhinoceroses in Kenya. *Scientia Parasitologica*, 12(2), 103–107. | Kenya |
| Ogore, P., Baker, R., Kenyanjui, M., &amp; Thorpe, W. (1999). Assessment of natural Ixodid tick infestations in sheep. *Small Ruminant Research*, 33(2), 103–107. | Kenya |
| Oguge, N.O., Durden, L.A., Keirans, J.E., Balami, H.D., & T.G. Schwan. (2009). Ectoparasites (sucking lice, fleas, and ticks) of small mammals in southeastern Kenya. *Medical and Veterinary Entomology* 23, 387–392. | Kenya |
| Oguge, N.O., RM, Ondiaka P. 1997. A preliminary survey of the macroparasite communities of rodents of Kahawa, central Kenya. *Belgian Journal of Zoology*, (Sup1) 127, 113–118 | Kenya |
| Omondi, D., Masiga, D. K., Fielding, B. C., Kariuki, E., Ajamma, Y. U., Mwamuye, M. M., ... & Villinger, J. (2017). Molecular detection of tick-borne pathogen diversities in ticks from livestock and reptiles along the shores and adjacent islands of Lake Victoria and Lake Baringo, Kenya. *Frontiers in Veterinary Science*, 4, 73. | Kenya |
| Ong'are, J.O., Munyua, W.K., Wilson, A.J., & F.G.R. Rinkanya. (1985). Survey of Tick Resistance in Kiambu District of Kenya: A Comparison of Two Resistance Test Methods. *Bulletin of Animal Health and Production in Africa*. 33, 89–99. | Kenya |
| Oswe, M., Odhiambo, R., Mutai, B., Nyakoe, N. Awinda, G., & J.N. Waitumbi. (2018). Zoonotic Pathogens in Ticks Collected from Livestock in Kenya. *Open Journal of Preventive Medicine* 8, 248–259. | Kenya |
| Peirce M. A. (1972). Rickettsia-like organisms in the blood of Turdus abyssinicus in Kenya. *Journal of Wildlife Diseases*, 8(3), 273–274. | Kenya |
| Pester, F. R. N., & Laurence, B. R. (1974). The parasite load of some African game animals. *Journal of Zoology*, 174(3), 397–406. | Kenya |
| Price, J. E., & Karstad, L. H. (1980). Free-living jackals (*Canis mesomelas*)-potential reservoir hosts for *Ehrlichia canis* in Kenya. *Journal of Wildlife Diseases*, 16(4), 469–473. | Kenya |
| Punyua, D. K., & Newson, R. M. (1985). The brown ear tick *Rhipicephalus appendiculatus* Neumann (Acarina: Ixodidae) and associated tick species on wild and domestic hosts at Muguga, Kenya. *The Journal of Parasitology*, 71(2), 248–252. | Kenya |
| Rinkanya, FGR, Kiniiya, HSN, (1992). Evaluation of the Efficacy of Ectopor *Rhipicephalus appendiculatus* ticks infesting cattle in Kenya. *Bulletin of Animal Health and Production in Africa*. p 197–200. | Kenya |
| Roberts, J. I. (1935). The ticks of rodents and their nests, and the discovery that *Rhipicephalus sanguineus* Latr. is the vector of tropical typhus in Kenya. *Epidemiology & Infection*, 35(1), 1–22. | Kenya |
| Rothen, J., Githaka, N., Kanduma, E.G., Olds, C., Pfluger, V., Mwaura S., Bishop, R.P., & C. Daubenberger. (2016). Matrix-assisted laser desorption/ionization time of flight mass spectrometry for comprehensive indexing of East African ixodid tick species. *Parasites & Vectors,* 9 (1), 151. | Kenya |
| Rutagwenda, T. (1984). A study of important camel diseases in northern Kenya with special emphasis on their control. *Camel Newsletter*, 1, 12–16. | Kenya |
| Sang, R. *et al.* (2006). Tickborne Arbovirus Surveillance in Market Livestock, Nairobi, Kenya. *Emerging Infectious Diseases* 12(7), 1074–1080. | Kenya |
| Sang, R., et al. (2011). Crimean-Congo Hemorrhagic Fever virus in Hyalommid Ticks, Northeastern Kenya. *Emerging Infectious Diseases* 17(8), 1502–1505. | Kenya |
| Solberg, I. M., & Aloo, I. A. (1976). Viral Isolates from Ixodid Ticks of Wild Animals in Kenya. *Wildlife Diseases*, 413–421. | Kenya |
| Tatchell, R.J., Chimwani, D., Chirchiru, S.J., Ong'are, J.O., Mwangi, E., Rinkanya, F., Whittington, D. (1986). A study of the justification for intensive tick control in Kenyan rangelands. *Veterinary Record*, 119, 401–403. | Kenya |
| Titcomb, G., Allan, B. F., Ainsworth, T., Henson, L., Hedlund, T., Pringle, R. M., ... & Young, H. S. (2017). Interacting effects of wildlife loss and climate on ticks and tick-borne disease. *Proceedings of the Royal Society B: Biological Sciences*, 284(1862), 20170475. | Kenya |
| Tomlinson, J. A., Horak, I. G., & Apanaskevich, D. A. (2018). Identity of *Haemaphysalis* (*Rhipistoma*) *muhsamae* Santos Dias, 1954 (Acari: Ixodidae) and *H*.(*R*.) *subterra* Hoogstraal, El Kammah & Camicas, 1992, parasites of carnivores and rodents in eastern and southern Africa. *Systematic Parasitology*, *95*(7), 673–691. | Kenya, Tanzania |
| Walker, A. R., Young, A. S., & Leitch, B. L. (1981). Assessment of *Theileria* infections in *Rhipicephalus appendiculatus* ticks collected from the field. *Zeitschrift für Parasitenkunde*, 65, 63–69. | Kenya |
| Walton, G.A. (1950). Relapsing fever in the Meru district of Kenya.*The East African Medical Journal*, 27(2), 94–98. | Kenya |
| Walton, G.A. (1955). Relapsing fever in the Digo district of Kenya colony. *The East African Medical Journal*, 32(10), 377–393 | Kenya |
| Wanjohi, J.M., Ngeranwa, J.N., Rumberia, R.M., Muraguri, G.R., & S.K. Mbogo. (2001). Immunization of cattle against East Coast fever using *Theileria parva* (Marikebuni) and relaxation of tick control in North Rift, Kenya. *Onderstepoort Journal of Veterinary Research,* 68, 217–223. | Kenya |
| Wanzala, W., Hassanali, A., Mukabana, W.R., Takken, W. (2018). Essential oils of indigenous plants protect livestock from infestations of *Rhipicephalus appendiculatus* and other tick species in herds grazing in natural pastures in western Kenya. *Journal of Pest Science*. 91, 395–404. | Kenya |
| Wanzala, W., & Okanga, S. (2006). Ticks (Acari: Ixodidae) associated with wildlife and vegetation of Haller park along the Kenyan coastline. *Journal of Medical Entomology*, 43(5), 789–794. | Kenya |
| Wesonga, F. D., Kitala, P. M., Gathuma, J. M., Njenga, M. J., & Ngumi, P. N. (2010). An assessment of tick-borne diseases constraints to livestock production in a smallholder livestock production system in Machakos District, Kenya. *Livestock Research for Rural Development* 22(6). | Kenya |
| Wesonga, F. D., Orinda, G. O., Ngae, G. N., & Grootenhuis, J. (2006). Comparative tick counts on game, cattle and sheep on a working game ranch in Kenya. *Tropical Animal Health and Production*, 38, 35–42. | Kenya |
| Wesonga, F. D., Wesongah, J. O., Chemuliti, J., Wanjala, K., Munga, L., & Gitau, P. (2006). Seroprevalence of *Ehrlichia ruminantium* (heartwater) in small ruminants in a pastoral production system in Narok District, Kenya. *Bulletin of Animal Health and Production in Africa*, 54(1), 23–33. | Kenya |
| Wilson, Von A.J., Schwarts, H.J., Dolan, R., Field, C.R., Rottcher, D. (1982). Epidemiological aspects of important diseases of camels in selected areas of Kenya. *Der Praktische Tierarzt*, 63(11), 974–985. | Kenya |
| Young AS, De Castro JJ, Burns C, Murphy DL. (1985). Potential of ear tags impregnated with acaricides for control of the brown ear tick (*Rhipicephalus appendiculatus*) infesting cattle. *Parasitology*. 90(2), 391–399. | Kenya |
| Young, A. S., Leitch, B. L., Newson, R. M., & Cunningham, M. P. (1986). Maintenance of *Theileria parva parva* infection in an endemic area of Kenya. *Parasitology*, 93 (1), 9–16. | Kenya |
| Young, A.S., Brown, C.G., Burridge, J.G., Gootenhuis, J.G., Kanhai, G.K., Purnell, R.E., Stagg, D.A. (1978). The incidence of theilerial parasites in East African buffalo (*Syncerus caffer*). *Tropenmedizin und Parasitologie*, 29(3), 281–288 | Kenya |
| Young, A.S., Leitch, B.L., Dolan, T.T., Mbogo, S.K., Ndungu, S.G., Grootenhuis, J.G., & J.J. De Castro. (1990). Evaluation of Infection and Treatment Methods in Immunization of Improved Cattle Against Theileriosis in an Endemic Area of Kenya. *Veterinary Parasitology* 35, 239–257. | Kenya |
| Young, A.S., Mutugi, J.J., Kariuki, D.P., Lampard, D., Maritim, A.C., Ngumi, P.N., Linyonyi, A., Leitch, B.L., Ndungu, S.G., Lesan, A.C., Mining, S.K., Grootenhuis, J.G., Orinda, G.O., & D. Wesonga. (1992). Immunisation of cattle against theileriosis in Nakuru District of Kenya by infection and treatment and the introduction of unconventional tick control. *Veterinary Parasitology,* 42, 225–240. | Kenya |
| Braae, U. C., Ngowi, H. A., & Johansen, M. V. (2013). Smallholder pig production: prevalence and risk factors of ectoparasites. *Veterinary parasitology*, *196*(1-2), 241–244. | Tanzania |
| Cutler, S. J., Browning, P., & Scott, J. C. (2006). Ornithodoros moubata, a soft tick vector for Rickettsia in east Africa?. *Annals of the New York Academy of Sciences*, 1078(1), 373–377. | Tanzania |
| Cutler, S. J., Jones, S. E., Wright, D. J. M., & Zhang, H. (2000). Cultivation of East African relapsing fever *Borrelia* and review of preceding events. *Journal of Spirochetal and Tick Borne Diseases*, 7(Fall/Win), 52–58. | Tanzania |
| Easton, E. R., & Tatchell, R. J. (1981). Field studies involving ticks of cattle and wild animals in the Sukumaland area of Tanzania, 1973-1976. In *Tick biology and control: proceedings of an International Conference January 27-29, 1981/edited by GB Whitehead and JD Gibson*. Grahamstown, South Africa: Tick Research Unit, Rhodes University, | Tanzania |
| Fukunaga, M., Ushijima, Y., Aoki, Y., & Talbert, A. (2001). Detection of Borrelia duttonii, a tick-borne relapsing fever agent in central Tanzania, within ticks by flagellin gene-based nested polymerase chain reaction. *Vector Borne and Zoonotic Diseases*, *1*(4), 331–338. | Tanzania |
| Fyumagwa, R. D., Hoare, R., Simmler, P., Meli, M. L., Hofmann-Lehmann, R., & Lutz, H. (2011). Molecular detection of *Anaplasma*, *Babesia* and *Theileria* species in a diversity of tick species from Ngorongoro Crater, Tanzania. *South African Journal of Wildlife*, 41(1), 79–86. | Tanzania |
| Fyumagwa, R. D., Runyoro, V., Horak, I. G., & Hoare, R. (2007). Ecology and control of ticks as disease vectors in wildlife of the Ngorongoro Crater, Tanzania. *South African Journal of Wildlife Research-24-month delayed open access*, *37*(1), 79–90. | Tanzania |
| Fyumagwa, R. D., Simmler, P., Meli, M. L., Hoare, R., Hofmann-Lehmann, R., & Lutz, H. (2009). Prevalence of Anaplasma marginale in different tick species from Ngorongoro Crater, Tanzania. Veterinary parasitology, 161(1-2), 154–157. | Tanzania |
| Geigy, E., & Mooser, H. (1955). Studies on the epidemiology of African relapsing fever in Tanganyika. *Journal of Tropical Medicine and Hygiene*, 58(9), 199–201. | Tanzania |
| Hoogstraal, H., Kaiser, M. N., & Easton, E. R. (1976). *Ornithodoros* (*Alectorobius*) *capensis* Neumann (Ixodoidea: Argasidae) parasitizing a human and birds nesting on islands in East African lakes. *Journal of Medical Entomology*, *12*(6), 703–704. | Tanzania |
| Keirans, J. E., Clifford, C. M., Hoogstraal, H., & Easton, E. R. (1976). Discovery of Nuttalliella namaqua Bedford (Acarina: Ixodoidea: Nuttalliellidae) in Tanzania and redescription of the female based on scanning electron microcopy. *Annals of the Entomological Society of America*, *69*(5), 926–932. | Tanzania |
| Kerario, I. I., Muleya, W., Chenyambuga, S., Koski, M., Hwang, S. G., & Simuunza, M. (2017). Abundance and distribution of Ixodid tick species infesting cattle reared under traditional farming systems in Tanzania. *African Journal of Agricultural Research*, 12(4), 286–299. | Tanzania |
| Kimbita, E. N. (2015). First report of *Rhipicephalus appendiculatus*, Echidnophaga gallinacea and Ctenocephalides felis on African pygmy hedgehogs (*Atelerix albiventris*) captured in Morogoro, Tanzania. *Research Opinions in Animal and Veterinary Sciences*, *5*(8), 329–334. | Tanzania |
| Kusiluka, L. J. M., Kambarage, D. M., Matthewman, R. W., Daborn, C. J., & Harrison, L. J. S. (1995). Prevalence of ectoparasites of goats in Tanzania. *Journal of Applied Animal Research*, 7(1), 69–74. | Tanzania |
| Kwak, Y. S., Kim, T. Y., Nam, S. H., Lee, I. Y., Kim, H. P., Mduma, S., ... & Yong, T. S. (2014). Ixodid tick infestation in cattle and wild animals in Maswa and Iringa, Tanzania. *The Korean journal of Parasitology*, *52*(5), 565. | Tanzania |
| Laisser, E. L. K., Chenyambuga, S. W., Karimuribo, E. D., Msalya, G., Kipanyula, M. J., Mwilawa, A. J., ... & Gwakisa, P. S. (2016). *Tick burden and acquisition of immunity to Theileria parva by Tarime cattle in comparison to Sukuma cattle under different tick control regimes in the Lake Zone of Tanzania*. 8(3), 21–28. | Tanzania |
| Laisser, E. L. K., Kipanyula, M. J., Msalya, G., Mdegela, R. H., Karimuribo, E. D., Mwilawa, A. J., ... & Chenyambuga, S. W. (2014). Tick burden and prevalence of Theileria parva infection in Tarime zebu cattle in the lake zone of Tanzania. *Tropical Animal Health and Production*, 46(8), 1391–1396. | Tanzania |
| Lee, S., Kim, J. Y., Yi, M. H., Lee, I. Y., Fyumagwa, R., & Yong, T. S. (2019). Comparative microbiomes of ticks collected from a black rhino and its surrounding environment. *International Journal for Parasitology: Parasites and Wildlife*, *9*, 239–243. | Tanzania |
| Lynen, G., Zeman, P., Bakuname, C., Di Giulio, G., Mtui, P., Sanka, P., & Jongejan, F. (2007). Cattle ticks of the genera *Rhipicephalus* and *Amblyomma* of economic importance in Tanzania: distribution assessed with GIS based on an extensive field survey. *Experimental and Applied Acarology*, *43*(4), 303–319. | Tanzania |
| Mamiro, K. A., Magwisha, H. B., Rukambile, E. J., Ruheta, M. R., Kimboka, E. J., Malulu, D. J., & Malele, I. I. (2016). Occurrence of ticks in cattle in the new pastoral farming areas in Rufiji district, Tanzania. *Journal of Veterinary Medicine*, 3420245. | Tanzania |
| Mchinja, S. J., and E. P. Sirima. (1983). *Field trial on the effects of the herbicide MCPA on ixodid ticks*. *Tropical Pest Management*. 29 (2), 196–197. | Tanzania |
| Msami, H. M. (2001). An epidemic of East Coast fever on a dairy farm in eastern Tanzania. *Preventive Veterinary Medicine*, *49*(1-2), 55–60. | Tanzania |
| Nagagi, Y. P., Kimaro, E. G., & Temba, V. (2020). Practical application and the possible emergence of tick resistance to commonly used acaricides in various districts of Tanzania. *Development*, 32, 8. | Tanzania |
| Newson, R. M., Mella, P. N. P., & Franklin, T. E. (1973). Observations on the numbers of the tick Rhipicephalus appendiculatus on the ears of zebu cattle in relation to hierarchical status in the herd. Tropical Animal Health and Production, 5(4), 281–283. | Tanzania |
| Ogden, N. H., Gwakisa, P., Swai, E., French, N. P., Fitzpatrick, J., Kambarage, D., & Bryant, M. (2003). Evaluation of PCR to detect Theileria parva in field-collected tick and bovine samples in Tanzania. *Veterinary Parasitology*, *112*(3), 177–183 | Tanzania |
| Ogden, N. H., Swai, E., Beauchamp, G., Karimuribo, E., Fitzpatrick, J. L., Bryant, M. J., ... & French, N. P. (2005). Risk factors for tick attachment to smallholder dairy cattle in Tanzania. *Preventive Veterinary Medicine*, *67*(2-3), 157–170. | Tanzania |
| Parker, J., Plowright, W., & Pierce, M. A. (1969). The epizootiology of African swine fever in Africa. *The Veterinary Record*, *85*(24), 668–674. | Tanzania |
| Phipps, J. (1950). *Ornithodoros moubata* Murray in Tanganyika. *East African Medical Journal*, 27(12), 475–82. | Tanzania |
| Scott, J. C., Wright, D. J. M., & Cutler, S. J. (2005). Typing African relapsing fever spirochetes. *Emerging Infectious Diseases*, 11(11), 1722. | Tanzania |
| Senzota, R. B. M. (1992). Rodent ectoparasites in the Serengeti National Park, Tanzania. *Tropical Ecology*, *33*(1), 29–33. | Tanzania |
| Swai, E. S., Karimuribo, E. D., & Kyakaisho, P. (2007). Further evidence of occurrence of *Argas persicus* Oken 1881 in free-range village chickens in Tanzania. *Livestock Research for Rural Development*. 19(6). | Tanzania |
| Swai, E. S., Karimuribo, E. D., Rugaimukamu, E. A., & Kambarage, D. M. (2006). Factors influencing the distribution of questing ticks and the prevalence stimation of *T*. *parva* infection in brown ear ticks in the Tanga region, Tanzania. *Journal of Vector Ecology*, 31(2), 224–228. | Tanzania |
| Tatchell, R. J., & Easton, E. (1986). Tick (Acari: Ixodidae) ecological studies in Tanzania. Bulletin of Entomological Research, 76(2), 229–246. | Tanzania |
| Walker, J. B. (1955). *Rhipicephalus pulchellus* Gerstäcker 1873: a description of the larva and nymph with notes on the adults and on its biology. *Parasitology*, *45*(1-2), 95–98. | Tanzania |
| Walker, J. B. (1966). *Rhipicephalus reichenowi* Zumpt, 1943: a re-description of the male and female and descriptions of the nymph and larva, together with an account of its known hosts and distribution. *Parasitology*, *56*(3), 457–469. | Tanzania |
| Walker, J. B., & Wiley, A. J. (1959). *Rhipicephalus camelopardalis* n. sp.(Ixodoidea, Ixodidae), a new species of tick from East African giraffes. *Parasitology*, 49(3–4), 448–453. | Tanzania |
| Walton, G. A. (1953). *Ornithodorus moubata* in wart-hog and porcupine burrows in Tanganyika territory. *Transactions of the Royal Society of Tropical Medicine and Hygiene*, *47*(5), 410–411. | Tanzania |
| Wambura, P. N., Gwakisa, P. S., Silayo, R. S., & Rugaimukamu, E. A. (1998). Breed-associated resistance to tick infestation in *Bos indicus* and their crosses with *Bos taurus*. *Veterinary Parasitology*, *77*(1), 63–70. | Tanzania |
| Warwick, B. T., Bak, E., Baldassarre, J., Gregg, E., Martinez, R., Kioko, J., ... & Kiffner, C. (2016). Abundance estimations of ixodid ticks on Boran cattle and Somali sheep in Northern Tanzania. *International Journal of Acarology*, 42(1), 12–17. | Tanzania |
| Yeoman, G. H., Walker, J. B., Ross, J. P. J., & Docker, T. M. (1967). The ixodid ticks of Tanzania. A study of the zoogeography of the Ixodidae of an East African country. 215. | Tanzania |
| Tatchell, R. J., & Easton, E. (1986). Tick (Acari: Ixodidae) ecological studies in Tanzania. *Bulletin of Entomological Research*, *76*(2), 229–246. | Tanzania |
| Block, W. (1968). Ticks from waterbuck and warthog in the Queen Elizabeth National Park, Uganda. East African Wildlife Journal, 6(1), 140–141. | Uganda |
| Byaruhanga, C., Collins, N.E., Knobel, D., Kabasa, W., & Oosthusizen, M.C. (2015). Endemic status of tick-borne infections and tick species diversity among transhumant zebu cattle in Karamoja Region, Uganda: Support for control approaches. *Veterinary Parasitology: Regional Studies and Reports*, 1-2, 21–30. | Uganda |
| Heisch, RB. (1952). First Record or *Ornithodoros erraticus* (lucas) from Uganda, with some speculations on the origin or *Spirochaeta duttoni* Novy and Knapp. *East African Medical Journal*, 29(11), 477–479. | Uganda |
| Hoogstraal, H., & el-Kammah, K. M. (1972). Notes on African *Haemaphysalis* ticks. X. *H*. (*Kaiseriana*) *aciculifer* Warburton and *H*. (*K*.) *rugosa* Santos Dias, the African representatives of the Spinigera sugroup (Ixodoidea: Ixodidae). *The Journal of Parasitology*, 58(5), 960–978. | Kenya, Ethiopia, Uganda |
| Kaiser, M. N., Sutherst, R. W., & Bourne, A. S. (1982). Relationship between ticks and Zebu cattle in southern Uganda. *Tropical Animal Health and Production*, 14(2), 63–74. | Uganda |
| Kasaija, P.D., Contreras, M., Kabi, F., Mugerwa, S., & J. Fuente. (2020). Vaccination with Recombinant Subolesin Antigens Provides Cross-Tick Species Protection in *Bos indicus* and Crossbred Cattle in Uganda. *Vaccines* 8, 319. | Uganda |
| Kitaka, F. X., Oteng, A. K., & Kamya, E. P. (1970). Toxaphene-resistant ticks occurring on cattle in Uganda: *Boophilus decoloratus*, *Rhipicephalus evertsi* and *Rhipicephalus appendiculatus*. *Bulletin of Epizootic Diseases of Africa*, 18, 137–142. | Uganda |
| Magona, J. W., Walubengo, J., & Kabi, F. (2011). Response of Nkedi Zebu and Ankole cattle to tick infestation and natural tick-borne, helminth and trypanosome infections in Uganda. Tropical Animal Health and Production, 43, 1019–1033. | Uganda |
| Matthysse, J.G., Murray, H.C., & E.P. Kamya. (1969). Acaricide Trials Against Rhipicephalus appendiculatus Neum., *Amblyomma variegatum* (F.) and *Boophilus decoloratus* (Koch) (Ixodidae) on Cattle in Uganda. *Bulletin of Entomological Research,* 58(3), 465–485. | Uganda |
| Miyama, T., Byaruhanga, J., Okamura, I., Uchida, L., Muramatsu, Y., Mwebembezi, W., Vudriko, P., & K. Makita. (2020). Effect of chemical tick control practices on tick infestation and *Theileria parva* infection in an intensive dairy production region of Uganda. *Ticks & Tick-borne Diseases,* 11, 101438. | Uganda |
| Muhanguzi, D., Byaruhanga, J., Amanyire, W. (2020). Invasive cattle ticks in East Africa: morphological and molecular confirmation of the presence of *Rhipicephalus microplus* in south-eastern Uganda. *Parasites & Vectors*, 13, 165. | Uganda |
| Nakao, R., Qiu, Y., Igarashi, M., Magona, J.W., Zhou, L., Ito, K., & C. Sugimoto. (2013). High prevalence of spotted fever group rickettsiae in *Amblyomma variegatum* from Uganda and their identification using sizes of intergenic spacers. *Ticks & Tick-borne Diseases,* 4, 506–512. | Uganda |
| Nakao, R., Stromdahl, E. Y., Magona, J. W., Faburay, B., Namangala, B., Malele, I., ... & Sugimoto, C. (2010). Development of loop-mediated isothermal amplification (LAMP) assays for rapid detection of *Ehrlichia ruminantium*. *BMC Microbiology*, 10, 1–11. | Uganda |
| Ndekezi, C., Nkamwesiga, J., Ochwo, S., Kimuda, M. P., Mwiine, F. N., Tweyongyere, R., ... & Muhanguzi, D. (2019). Identification of ixodid tick-specific aquaporin-1 potential anti-tick vaccine epitopes: an in-silico analysis. *Frontiers in Bioengineering and Biotechnology*, 7, 236. | Uganda |
| Obara, I., Githaka, N., Nijhof, A., Krücken, J., Nanteza, A., Odongo, D., ... & Bishop, R. P. (2020). The *Rhipicephalus appendiculatus* tick vector of Theileria parva is absent from cape buffalo (*Syncerus caffer*) populations and associated ecosystems in northern Uganda. *Parasitology Research*, 119, 2363–2367. | Uganda |
| Okello-Onen, J., Tukahirwa, E. M., Perry, B. D., Rowlands, G. J., Nagda, S. M., Musisi, G., ... & Opuda-Asibo, J. (1999). Population dynamics of ticks on indigenous cattle in a pastoral dry to semi-arid rangeland zone of Uganda. *Experimental & Applied Acarology*, 23, 79–88. | Uganda |
| Okello-Onen, J., Tukahirwa, E.M., Perry, B.D., Rowlands, G.J., Nagda, S.N., et al. (2003). The Impact of Tick Control on the Productivity of Indigenous Cattle under Ranch Conditions in Uganda. Tropical Animal Health and Production, 35, 237–247. | Uganda |
| Proboste, T., Kalema-Zikusoka, G., Altet, L., Solano-Gallego, L., Fernández de Mera, I. G., Chirife, A. D., ... & Millán, J. (2015). Infection and exposure to vector-borne pathogens in rural dogs and their ticks, Uganda. *Parasites & Vectors*, 8, 1–9. | Uganda |
| Randolph, S. E., & Rogers, D. J. (1997). A generic population model for the African tick *Rhipicephalus appendiculatus*. *Parasitology*, 115(3), 265–279. | Uganda |
| Rubaire-Akiiki, C.M., Okello-Onen, J., Musunga, D., Kabagambe, E.K. Vaarst, M. et al. (2006). Effect of agro-ecological zone and grazing system on incidence of East Coast Fever in calves in Mbale and Sironko Districts of Eastern Uganda. *Preventive Veterinary Medicine*, 75, 251–266. | Uganda |
| Schuh, A.J., Amman, B.R., Apanaskevich, D.A. et al. (2016). No evidence for the involvement of the argasid tick *Ornithodoros* *faini*in the enzootic maintenance of Marburg virus within Egyptian rousette bats *Rousettus aegyptiacus*. *Parasites & Vectors,* 9(1) 1–3. | Uganda |
| Balinandi, S., Mugisha, L., Bbira, J., Kabasa, W., Nakayiki, T., Bakkes, D. K., ... & Malmberg, M. (2019). General and local morphological anomalies in *Amblyomma lepidum* (Acari: Ixodidae) and *Rhipicephalus decoloratus* infesting cattle in Uganda. *Journal of Medical Entomology*, 56(3), 873–877. | Uganda |
| Steyn, J.J. (1955).  A second locality record in Uganda for the tick parasite, *Hunterellus hookeri* Howard, and a few discussions on other tick parasites. *The East African Medical Journal*, 32(9), 357–360. | Uganda |
| Tayebwa, D. S., Vudriko, P., Tuvshintulga, B., Guswanto, A., Nugraha, A. B., Gantuya, S., ... & Igarashi, I. (2018). Molecular epidemiology of Babesia species, *Theileria parva*, and *Anaplasma marginale* infecting cattle and the tick control malpractices in Central and Eastern Uganda. *Ticks & Tick-borne Diseases*, 9(6), 1475–1483. | Uganda |
| Vudriko, P., Okwee-Acai, J., Tayebwa, D. S., Byaruhanga, J., Kakooza, S., Wampande, E., ... & Suzuki, H. (2016). Emergence of multi-acaricide resistant *Rhipicephalus* ticks and its implication on chemical tick control in Uganda. *Parasites & Vectors*, 9(1), 1–13. | Uganda |
| Vudriko, P., Okwee-Acai, J., Byaruhanga, J., Tayebwa, D. S., Okech, S. G., Tweyongyere, R., … Suzuki, H. (2018). Chemical tick control practices in southwestern and northwestern Uganda. Ticks & Tick-Borne Diseases, 9(4), 945–955. | Uganda |
| Vudriko, P., Umemiya-Shirafuji, R., Okwee-Acai, J., Tayebwa, D.S., Byaruhanga, J., et al. (2017). Genetic mutations in sodium channel domain II and carboxylesterase genes associated with phenotypic resistance against synthetic pyrethroids by *Rhipicephalus* (*Boophilus*) *decoloratus* ticks in Uganda. *Pesticide Biochemistry and Physiology*, 143, 181–190. | Uganda |
| Walker, J. B., Keirans, J. E., & Pegram, R. G. (1993). *Rhipicephalus aquatilis* sp. nov.(Acari: Ixodidae), a new tick species parasitic mainly on the sitatunga, *Tragelaphus spekei*, in East and Central Africa. 60 (3). 205–210. | Uganda |
